# Lipoprotein(a) and recurrent atherosclerotic cardiovascular events: the US Family Heart Database

**DOI:** 10.1093/eurheartj/ehaf297

**Published:** 2025-05-07

**Authors:** Diane E MacDougall, Anne Tybjærg-Hansen, Joshua W Knowles, Theresa P Stern, Bonnie K Hartsuff, Mary P McGowan, Seth J Baum, Katherine A Wilemon, Børge G Nordestgaard

**Affiliations:** Department of Research, Family Heart Foundation, 5548 First Coast Hwy, Fernandina Beach, FL 32034, USA; Department of Clinical Biochemistry, Copenhagen University Hospital—Rigshospitalet, Copenhagen, Denmark; Department of Clinical Medicine, Faculty of Health and Medical Sciences, University of Copenhagen, Copenhagen, Denmark; Division of Cardiovascular Medicine, Cardiovascular Institute and Prevention Research Center, Stanford University, Stanford, CA, USA; BIA Clinical Group, Ann Arbor, MI, USA; BIA Clinical Group, Ann Arbor, MI, USA; Department of Research, Family Heart Foundation, 5548 First Coast Hwy, Fernandina Beach, FL 32034, USA; Flourish Research, Apex, NC, USA; Department of Research, Family Heart Foundation, 5548 First Coast Hwy, Fernandina Beach, FL 32034, USA; Department of Clinical Medicine, Faculty of Health and Medical Sciences, University of Copenhagen, Copenhagen, Denmark; Department of Clinical Biochemistry, Copenhagen University Hospital–Herlev and Gentofte, Herlev, Denmark

**Keywords:** Lipoprotein(a), Recurrent atherosclerotic cardiovascular disease, Sex, Race, Ethnicity

## Abstract

**Background and Aims:**

Higher levels of lipoprotein(a) drive increasing risk of atherosclerotic cardiovascular disease (ASCVD) in otherwise healthy individuals regardless of sex and race/ethnicity. This study aimed to evaluate whether this is also true for recurrent ASCVD, and whether LDL cholesterol-lowering therapy possibly mitigates such a relationship.

**Methods:**

In US medical claims between 2012 and 2022 for 340 million individuals, 273 770 had diagnosed ASCVD and lipoprotein(a) measured in nmol/L. These women (*n* = 117 269; 43%) and men (*n* = 156 501; 57%) included Black (*n* = 22 451; 8%), Hispanic (*n* = 24 606; 9%), and White (*n* = 161 165; 59%) individuals.

**Results:**

Lipoprotein(a) levels were higher in women vs men and in Black vs Hispanic and White individuals. During a median follow-up of 5.4 years, 41 687 individuals (15%) experienced recurrent ASCVD. Higher lipoprotein(a) levels were associated with continuously increasing risk of recurrent ASCVD. Compared to individuals with lipoprotein(a) < 15 nmol/L, the adjusted hazard ratios for recurrent ASCVD events were 1.04 (95% confidence interval 1.01–1.07) for 15–79 nmol/L, 1.15 (1.12–1.19) for 80–179 nmol/L, 1.29 (1.25–1.33) for 180–299 nmol/L, and 1.45 (1.39–1.51) for ≥300 nmol/L. Results were similar for individual ASCVD components, and in sex, race/ethnicity, baseline ASCVD, and diabetes subgroups; however, high impact LDL cholesterol-lowering therapy possibly mitigates the deleterious effect of lipoprotein(a) ≥ 180 nmol/L, most pronounced in those on PCSK9 inhibitors. Interaction on recurrent ASCVD events between lipoprotein(a) categories and sex, race/ethnicity, baseline ASCVD, diabetes, and impact of LDL cholesterol-lowering therapy use had *P*-values of .61, .06, .33, .91, and 2 × 10^−8^, respectively.

**Conclusions:**

In 273 770 individuals with ASCVD, higher lipoprotein(a) levels were associated with continuously increasing risk of recurrent ASCVD events regardless of sex and race/ethnicity that may have been partially mitigated by high impact LDL cholesterol-lowering therapy.

## Structured Graphical Abstract

**Structured Graphical Abstract:**
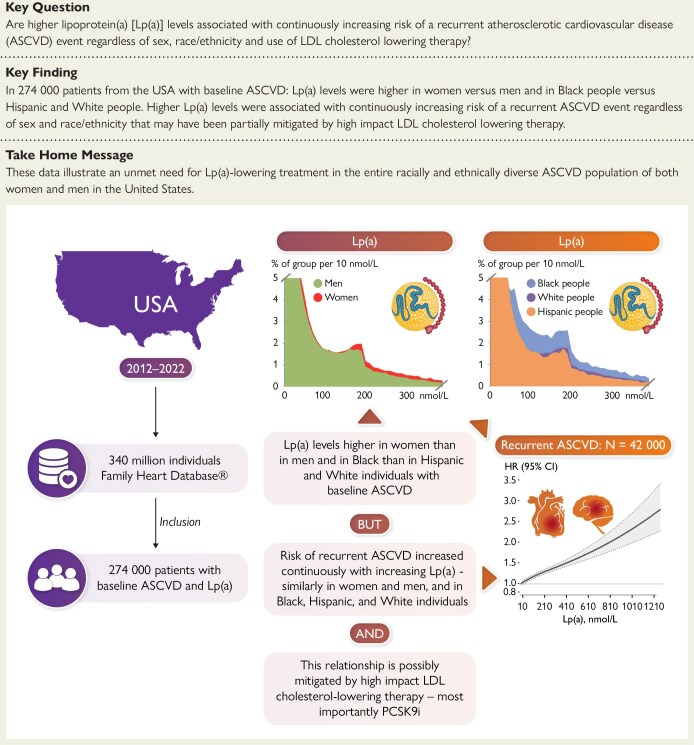

**See the editorial comment for this article ‘Ignorance is not bliss: the importance of lipoprotein(a) testing', by M.L. O’Donoghue and V. Monguillon, https://doi.org/10.1093/eurheartj/ehaf422.**

## Introduction

Higher lipoprotein(a) levels are a causal risk factor for continuously increased risk of an incident atherosclerotic cardiovascular disease (ASCVD) event in otherwise healthy individuals, regardless of sex and race/ethnicity.^1–5^ Whether this is also true for recurrent ASCVD events has not yet been determined. Although there are currently no drugs approved to lower lipoprotein(a), several investigational therapies which reduce lipoprotein(a) levels by 80%–98%, are being tested in individuals with ASCVD and lipoprotein(a) levels exceeding 175–200 nmol/L, aiming to reduce recurrent ASCVD events [ClinicalTrials.gov: NCT04023552 (ASCVD only), NCT05581303 (ASCVD only), and NCT06292013].^6–8^ Until such therapies become available, it is recommended to use high impact LDL cholesterol-lowering therapy in patients at high risk of ASCVD with very high levels of lipoprotein(a).^1–4^ Therefore, information on whether higher lipoprotein(a) levels drive continuously increased risk of a recurrent ASCVD event regardless of sex, race/ethnicity, and use of high impact LDL cholesterol-lowering therapy is needed.

We tested the hypothesis that higher lipoprotein(a) levels are associated with continuously increasing risk of a recurrent ASCVD event regardless of sex, race/ethnicity, and use of high impact LDL cholesterol-lowering therapy. Data from 273 770 US individuals in the Family Heart Database were analysed from 30 days post-ASCVD diagnosis until a recurrent ASCVD event as a function of higher lipoprotein(a) levels. This cohort is of sufficient size to assess the full range of lipoprotein(a) levels, particularly those exceeding 175–200 nmol/L included in ongoing randomized trials, and to detect any variation in the relationship by sex, race/ethnicity, and impact of LDL cholesterol-lowering therapy.

As lipoprotein(a) levels are >90% genetically determined and are largely stable throughout life,^4^ the relationship of high lipoprotein(a) levels with risk of recurrent ASCVD events should not be substantially affected by lifestyle confounding and reverse causation according to the principle of Mendelian randomization.^9,10^ Importantly, we evaluated the effect of sex and race/ethnicity which are known determinants of lipoprotein(a) levels,^1–5,11^ and therefore also adjusted for these variables in the analyses.

## Methods

### Study population

The Family Heart Database^12^ contains US medical claims from 340 million individuals between 2012 and 2022 that annually represents at least half of the national census population; corresponding laboratory (primarily lipid) data are also available in about one third of these individuals. Within the entire database, longitudinal medical claims of medication use, diagnoses, procedures, and/or surgeries are available over 1–3 years (79 445 324 individuals), 4–6 years (72 690 105 individuals), and 7–11 years (162 956 831 individuals), and some individuals have at least one lipoprotein(a) test measured in nmol/L (*n* = 1 232 206) or mg/dL (*n* = 46 059). Race/ethnicity was defined by self-report. The database was created and has been regularly updated by the Family Heart Foundation® since 2014, characterizing patterns of real-world medical encounters by US individuals with lipid disorders and cardiovascular risk. All data are sourced from Symphony Health, an ICON plc Company (Integrated Dataverse, IDV®, Blue Bell, PA, USA), and received de-identified per Health Insurance Portability and Accountability Act (HIPAA) standards. Standard coding systems are used for medications [National Drug Code (NDC)], diagnosis and procedures [International Statistical Classification of Diseases and Related Health Problems (ICD-9 and ICD-10), Current Procedural Terminology (CPT), and Healthcare Common Procedure Coding System (HCPCS)], and laboratory data [Logical Observation Identifiers Names and Codes (LOINC)].

Since this was an analysis of de-identified data (see above), neither informed consent nor ethics approval was required.

### Baseline atherosclerotic cardiovascular disease

All adults (≥18 years of age) with medical claim or lab result activity from May 2012 to December 2022 were evaluated; those included in the ASCVD cohort had established ASCVD, ≥ 1 lipoprotein(a) test measured in nmol/L, and ≥1 medical claim occurring at least 30 days after the date of established ASCVD diagnosis. Individuals met the definition of established ASCVD with ≥1 inpatient or ≥2 outpatient qualified diagnosis claims or ≥1 procedure or surgical medical claims corresponding to clinical conditions of myocardial infarction, other acute coronary syndrome, percutaneous coronary intervention (PCI), coronary artery bypass graft (CABG), stable angina, ischaemic stroke, other cerebral vascular disease, transient ischaemic attack, peripheral vascular disease, and general ASCVD (see Supplementary data online, *Table S1* for code list).

Many individuals met the definition of established ASCVD while hospitalized, including 12% who experienced an acute cardiovascular event (myocardial infarction, other acute coronary syndromes, ischaemic stroke, PCI, or CABG) and 32% who had inpatient reports of past or other current qualifying ASCVD events; 56% met the definition of established ASCVD with outpatient reports of current or past qualifying ASCVD events.

### Recurrent atherosclerotic cardiovascular disease events during follow-up

Recurrent ASCVD events during follow-up included hospitalization for myocardial infarction, other acute coronary syndromes, and ischaemic stroke or procedures/surgeries of PCI and CABG that were identified by qualified medical claims (≥1 inpatient diagnosis or procedure or surgery). Validation studies assessing identification of myocardial infarction and ischaemic stroke by medical claims vs chart review or clinical trial adjudication have demonstrated positive predictive values of 96% and 89%, respectively, with corresponding sensitivities of 83% and 67%.^13,14^ The follow-up period was from 30 days after the baseline ASCVD diagnosis until the date of last activity (medical claim or lab result) within the database. Secondary endpoints included time to first occurrence of each component: myocardial infarction, other acute coronary syndrome, ischaemic stroke, PCI, or CABG events.

### Lipoprotein(a) and other lipid measurements

All lipoprotein(a) testing used an immunoturbidimetric assay according to industry standard procedures and reported results in nmol/L. Lipoprotein(a) testing was completed by multiple vendors, with more than 75% of measures confirmed to have used standardized instrumentation, calibration, and reagents, an analytical measurement range of 10–200 nmol/L without dilution, and a reportable range of 10–600 nmol/L after dilution. All values < 10 nmol/L and non-nominal values > 600 nmol/L were excluded from restricted cubic splines examining lipoprotein(a) on a continuous scale; however, they were included in the main analyses using clinically relevant categories of lipoprotein(a) levels. For everyone included in the analyses, the lowest of all available lipoprotein(a) values in nmol/L was used.

For other lipid parameters (total cholesterol, LDL cholesterol, and total triglycerides), the median of all values assessed from 60 days after the baseline ASCVD through end of follow-up was used in statistical analyses, to achieve the best possible adjustment for levels of these lipid parameters during the entire follow-up period.

### Other covariates for adjustment and subgroups

ICD-9 and ICD-10 codes (see Supplementary data online, *Table S1*) were used to confirm the presence of comorbidities of diabetes, hypertension, chronic kidney disease, atrial fibrillation, familial hypercholesterolaemia and hyperlipidaemia. An updated Charlson Comorbidity Index with associated code list was also applied.^15^ Comorbidities were assessed from earliest activity to 365 days after the baseline ASCVD. The use of select LDL cholesterol-lowering therapy and combinations [statins, proprotein convertase subtilisin/kexin type 9 inhibitors (PCSK9i); ezetimibe and bempedoic acid], duration of use of LDL cholesterol-lowering therapy, use of anti-hypertension therapy, and use of anti-platelet therapy were assessed from 8 to 365 days after the baseline ASCVD. Self-reports of education level, household income, and US region were also included.

Subgroup analyses were performed by sex (women and men), self-reported race/ethnicity (Black, Hispanic, and White individuals), baseline ASCVD (coronary, cerebrovascular and peripheral), baseline diabetes (yes/no) and LDL cholesterol-lowering therapy use (high impact, low/moderate impact, and none; see *Table 1*). Individuals were included in a single category according to the most impactful LDL cholesterol-lowering therapy for which a paid prescription was filled during the medication assessment period (Days 8–365).

**Table 1:** Impact of LDL cholesterol-lowering drugs and combinations

	High impact	Low/moderate impact	No use
PCSK9i mono- or combination therapy	X		
Statin + ezetimibe, statin + bempedoic acid	X		
High intensity statin monotherapy	X		
Low/moderate intensity statin monotherapy		X	
Bempedoic acid and/or ezetimibe		X	
None			X

Mono- and combination therapies are listed in descending order of impact. Individuals are included in a single category according to the most impactful therapy used based on paid prescription fill during the medication assessment period (Days 8–365).

PCSK9i, proprotein convertase subtilisin/kexin type 9 inhibitors.

### Statistical analyses

The primary endpoint was time to first occurrence of a recurrent ASCVD event during the follow-up period. The association between lipoprotein(a) levels and recurrent ASCVD events during follow-up was estimated using a Cox proportional hazard model. For individuals experiencing at least one recurrent event, time to the first event was used for the analysis; for individuals not experiencing a recurrent ASCVD event, time was censored at the end of follow-up. The unadjusted Cox model included time to first recurrent event as the dependent variable and lipoprotein(a) levels as the independent variable. The model adjusted for sex, race/ethnicity, and age is considered our main analysis, as lipoprotein(a) is >90% genetically determined,^4^ and therefore unlikely to be confounded by lifestyle confounding factors or reverse causation according to the principle of Mendelian randomization.^9,10^ However, we also performed multivariable adjustment for all covariates. These additional independent variables (age; sex; race/ethnicity; education level; household income; US region; hyperlipidaemia; hypertension; chronic kidney disease; diabetes; atrial fibrillation; baseline ASCVD status; use of LDL-lowering therapy, anti-hypertension therapy and anti-platelet therapy; duration of use of LDL cholesterol-lowering therapy, Charlson Comorbidity Index; LDL cholesterol; total triglycerides; and total cholesterol) were added one-by-one and in various combinations to the Cox model. For additional details regarding adjustment for covariates, see Supplementary data online, *Table S2*. Lipoprotein(a) was included in the analysis two different ways: categorized and continuous. The categorized values were based on clinically meaningful, approximate individual percentile distribution with a focus on the extreme high levels: <15 nmol/L (1st–32nd percentiles; reference); 15–79 nmol/L (33rd–66th percentiles); 80–179 nmol/L (67th–84th percentiles); 180–299 nmol/L (85th–94th percentiles); and ≥300 nmol/L (95th–100th percentiles). Continuous lipoprotein(a) values were included in the analysis adjusted for sex, race/ethnicity, and age using restricted cubic splines.^16^

Similar Cox analyses using categorized lipoprotein(a) and adjusted for sex, race/ethnicity, and age were performed to analyse time to first occurrence of each of the component events that comprise recurrent ASCVD.

Subgroup analyses with sufficient statistical power for meaningful results were performed using a Cox proportional hazard model adjusted for sex, race/ethnicity, and age with categorized lipoprotein(a) by sex (men/women), by the three major race/ethnicity categories (Black, Hispanic, and White individuals), by baseline ASCVD categories (coronary, cerebrovascular, and peripheral), by baseline diabetes (yes/no), and by impact of LDL cholesterol-lowering therapy (high impact, low/moderate impact, or none used). Additional Cox analyses were performed for the subgroups of sex and race/ethnicity using continuous lipoprotein(a); and within the subgroup of high impact LDL cholesterol-lowering therapy comparing PCSK9i use (yes/no) using categorized lipoprotein(a). Finally, one more analysis was performed using Poisson regression to assess the absolute rate of recurrent ASCVD events by lipoprotein(a) levels and LDL cholesterol-lowering therapy use. First recurrent events per 1000 person-years were counted within categories defined by lipoprotein(a) (<80, 80–179, ≥180 nmol/L) and LDL cholesterol-lowering therapy use (high impact, low/moderate impact, none used) with log of the time in years from baseline to first recurrent ASCVD event or, if no recurrent ASCVD event, end of follow-up.

Within the full ASCVD cohort, the interactions on recurrent ASCVD events between lipoprotein(a) categories and variables of sex, race/ethnicity, baseline ASCVD, baseline diabetes and LDL cholesterol-lowering therapy use were formally tested by calculating the difference in log likelihoods from the analyses with and without the interaction terms in the model. Additionally, within each sex and race/ethnicity subgroup, corresponding race/ethnicity or sex interactions were assessed. Finally, within the high impact LDL-C-lowering therapy subgroup, the interaction of PCSK9i use was assessed. Sensitivity analyses were performed using a second categorization of lipoprotein(a) levels based on guidance from the US National Lipid Association:^2^ < 75 nmol/L; 75–124 nmol/L; 125–199 nmol/L; 200–399 nmol/L; and ≥400 nmol/L.

All statistical analyses were performed using SAS version 9.4.

## Results

### Study population

There were 273 770 individuals with baseline established ASCVD across five lipoprotein(a) categories ranging from <15 (*n* = 85 025) to ≥300 nmol/L (*n* = 14 006) (*Table 2*). From lowest to highest lipoprotein(a) category, a progressively higher percentage of women (from 38% to 54%) and Black individuals (from 5% to 15%) was observed, with a corresponding progressively lower percentage of men (from 62% to 46%), Hispanic (from 10% to 7%), and White (from 63% to 55%) individuals (*Table 2*; see corresponding Supplementary data online, *Tables S3*–*S8* in women, men, and Black, Hispanic, and White individuals). This was expected, as lipoprotein(a) levels differ by sex and race/ethnicity.^1,4,11^ However, other potential confounders were largely similar by levels of lipoprotein(a), again as expected as lipoprotein(a) levels are >90% genetically determined.^4^ As expected, LDL cholesterol levels and the use of LDL cholesterol-lowering therapy were higher with higher lipoprotein(a) levels, as lipoprotein(a) cholesterol is included in the LDL cholesterol measurement making it more likely that clinicians will prescribe lipid-lowering therapy. The use of PCSK9i was low (1%).

**Table 2:** Baseline characteristics and comorbidities by lipoprotein(a) category in individuals with ASCVD at baseline

	Lipoprotein(a) category (nmol/L and percentiles)				
	<15 <33% (*n* = 85 025)	15–79 33%–66% (*n* = 97 958)	80–179 67%–84% (*n* = 47 240)	180–299 85%–94% (*n* = 29 541)	≥300 ≥95% (*n* = 14 006)
Age, years, median (IQR)	64 (55–70)	64 (56–70)	63 (55–70)	63 (55–70)	64 (56–70)
Women, *n* (%)	32 400 (38)	42 625 (44)	20 524 (43)	14 208 (48)	7512 (54)
Race/ethnicity, *n* (%)					
Black individuals	3895 (5)	7281 (7)	5691 (12)	3510 (12)	2074 (15)
Hispanic individuals	8276 (10)	9216 (9)	3919 (8)	2233 (8)	962 (7)
White individuals	53 515 (63)	56 831 (58)	26 270 (56)	16 898 (57)	7651 (55)
Other individuals	3651 (4)	5298 (5)	2154 (5)	1145 (4)	537 (4)
Unknown	15 688 (18)	19 332 (20)	9206 (19)	5755 (19)	2782 (20)
Baseline ASCVD, *n* (%)					
Coronary	57 274 (67)	65 024 (66)	31 834 (67)	20 432 (69)	9901 (71)
Cerebrovascular	22 401 (26)	26 534 (27)	12 690 (27)	7784 (26)	3523 (25)
Peripheral	16 066 (19)	19 659 (20)	9196 (19)	5500 (19)	2742 (20)
Unknown	1389 (2)	1420 (1)	654 (1)	402 (1)	144 (1)
Charlson Comorbidity Index, *n* (%)					
0	38 501 (45)	43 557 (44)	21 363 (45)	13 601 (46)	6263 (45)
1–2	28 866 (34)	33 366 (34)	16 000 (34)	9906 (34)	4787 (34)
3+	17 658 (21)	21 035 (21)	9877 (21)	6034 (20)	2956 (21)
Risk factors and drug use, *n* (%)					
Hypertension	63 634 (75)	73 518 (75)	35 611 (75)	22 478 (76)	10 965 (78)
Treated w/ drugs	43 954 (52)	50 577 (52)	24 678 (52)	15 863 (54)	7874 (56)
Diabetes	28 318 (33)	31 680 (32)	15 586 (33)	9508 (32)	4979 (36)
Familial	593 (0.7)	751 (0.8)	396 (0.8)	307 (1.0)	154 (1.1)
Hypercholesterolaemia					
LDL-C-lowering drugs, *n* (%)					
High impact	24 876 (29)	28 962 (30)	15 316 (32)	11 160 (38)	6178 (44)
PCSK9i	1051 (1)	1076 (1)	732 (2)	566 (2)	356 (3)
Low/moderate impact	23 560 (28)	26 795 (27)	12 524 (27)	7442 (25)	3114 (22)
None	36 589 (43)	42 201 (43)	19 400 (41)	10 939 (37)	4714 (34)
Duration of use > 75%	28 097 (33)	32 114 (33)	15 720 (33)	10 885 (37)	5532 (39)
Prescribed anti-platelet drugs	18 857 (22)	22 091 (23)	11 666 (25)	7822 (26)	4139 (30)
Laboratory values, median [IQR]					
Lipoprotein(a), nmol/L	10 (10–10)	34 (22–51)	128 (102–155)	217 (194–253)	365 (329–426)
LDL cholesterol^a^, mg/dL	78 (59–105)	81 (62–110)	83 (64–111)	83 (66–110)	88 (71–112)
Triglycerides^a^, mg/dL	115 (83–163)	109 (80–150)	104 (77–143)	106 (79–144)	107 (81–147)

Individuals may be included in multiple baseline ASCVD categories. Comorbidities were assessed from the earliest activity in the database to 365 days after diagnosis of ASCVD. Drug use was determined from 8 to 365 days after diagnosis of ASCVD. LDL cholesterol-lowering drugs were categorized according to *Table 1*. The lowest of all available lipoprotein(a) values in nmol/L was used. LDL cholesterol and triglyceride values were median of values within 60 days after diagnosis of ASCVD through end of follow-up.

ASCVD, atherosclerotic cardiovascular disease; IQR, interquartile range [25th, 75th percentiles]; PCSK9i, proprotein convertase subtilisin/kexin type 9 inhibitors.

^a^64% and 74% of individuals had values for LDL cholesterol and triglycerides, respectively, within this time period.

**Figure 1:**
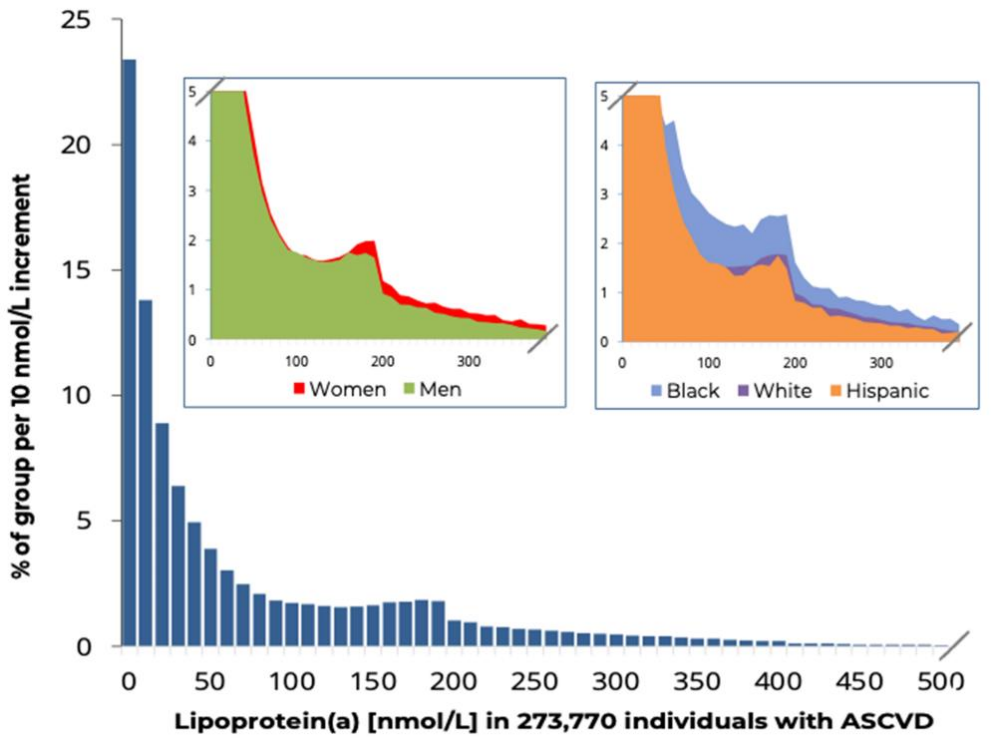
Distribution of lipoprotein(a) in individuals with ASCVD at baseline and by sex and race/ethnicity. Lipoprotein(a) values > 500 nmol/L and in stratified analysis > 400 nmol/L are not displayed. The highest percentile of lipoprotein(a) values (*n* = 2762 individuals) ranged from 453 to 1153 nmol/L. ASCVD, atherosclerotic cardiovascular disease

The distribution of lipoprotein(a) levels in all individuals with baseline ASCVD was skewed with a tail towards very high levels, which was also the case in subgroups stratified by sex and race/ethnicity (*Figure 1*); these results were similar when those on PCSK9i were excluded (data not shown). Levels were higher in women than in men, and higher in Black than in Hispanic and White individuals. The slightly higher levels of lipoprotein(a) before and after the value of 200 nmol/L likely is because many assays have a dilution cut-off around this value, meaning that measurements above vs below the value of 200 nmol/L may differ slightly in accuracy.

### Continuous lipoprotein(a) and recurrent atherosclerotic cardiovascular disease events

During a median follow-up of 5.4 years (30 days to 10.6 years; 1 511 210 person-years), 41 687 individuals experienced a recurrent ASCVD event, including 15 078 women, 26 609 men, and 4064 Black, 3784 Hispanic, and 24 139 White individuals.

**Figure 2:**
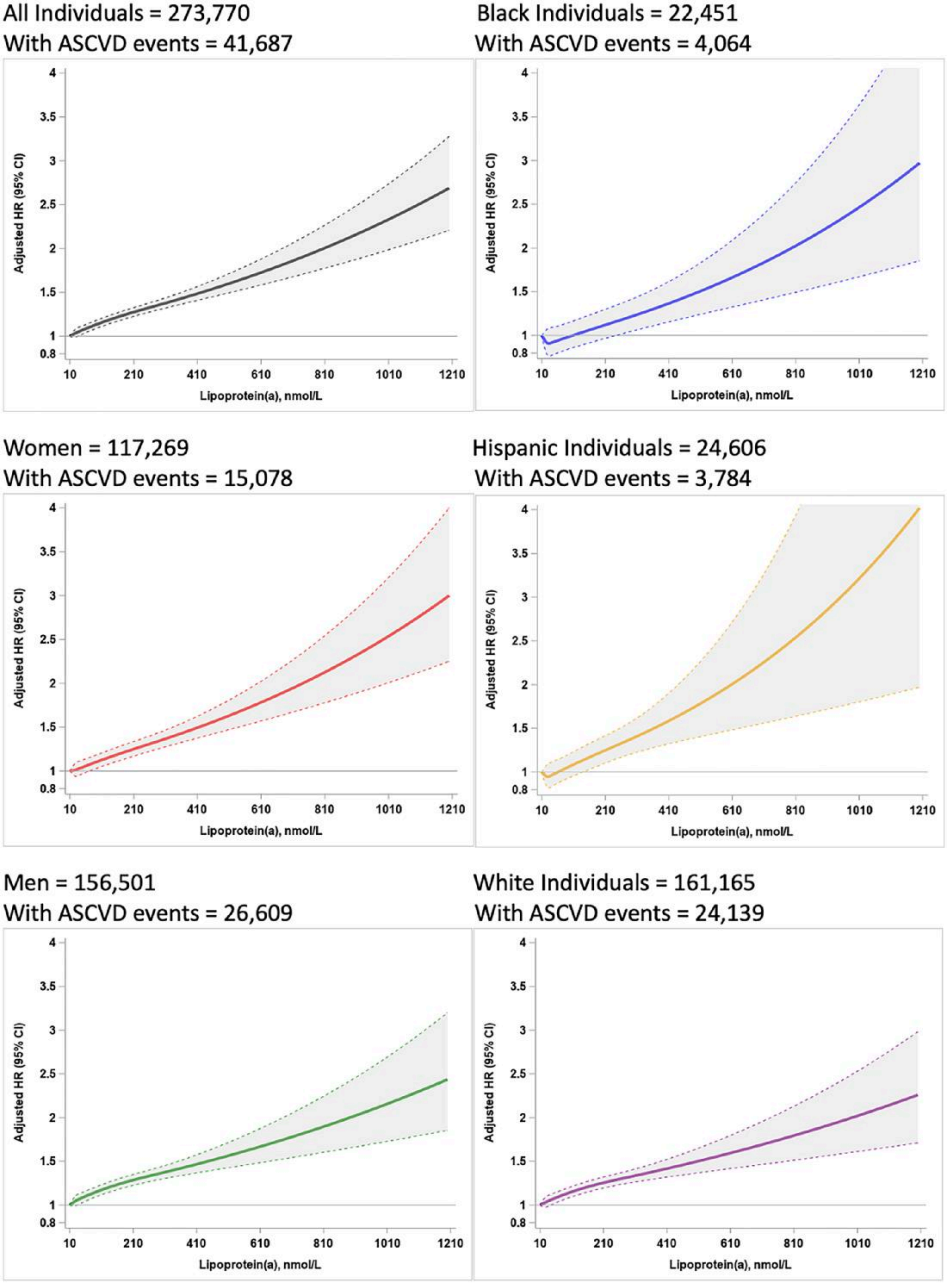
Risk of recurrent ASCVD events as a function of continuously higher lipoprotein(a) levels. Cox proportional hazards analyses with time to recurrent ASCVD event following initial ASCVD at baseline as the dependent variable and lipoprotein(a) continuously as the independent variables. Hazard ratios adjusted for sex, race/ethnicity, and age are shown as filled lines while 95% CIs are shown as shaded areas. When the lower 95% CI no longer crosses the hazard ratio of 1.0 shown as a grey line, the hazard ratio is significantly different from 1.0. Lipoprotein(a) levels < 10 nmol/L and non-nominal levels reported as ‘>600’ are excluded. ASCVD, atherosclerotic cardiovascular disease; CI, confidence interval; HR, hazard ratio

Higher lipoprotein(a) levels were associated with continuously increasing risk of a recurrent ASCVD event overall, and in stratified analyses of women, men, and Black, Hispanic, and White individuals (*Figure 2*). Confidence intervals (CIs) around adjusted hazard ratios are wider in Black and Hispanic individuals than in White individuals due to smaller numbers of individuals in those race/ethnicity subgroups.

### Lipoprotein(a) categories and recurrent atherosclerotic cardiovascular disease events

**Figure 3:**
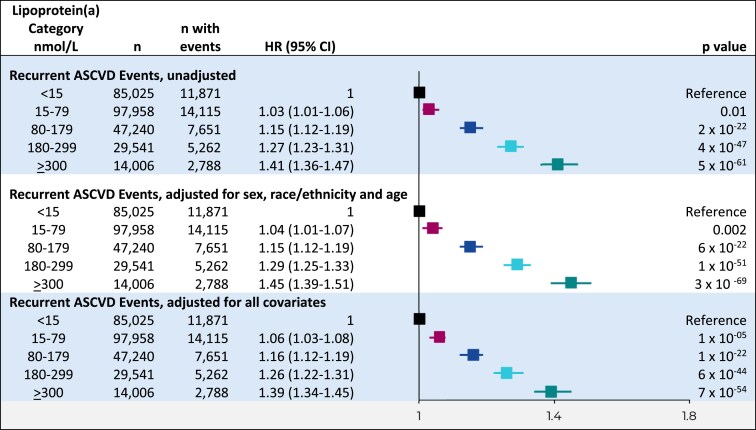
Risk of recurrent ASCVD events by clinically relevant categories of lipoprotein(a). Cox proportional hazards analyses with time to recurrent ASCVD event following initial ASCVD at baseline as the dependent variable and lipoprotein(a) categories as the independent variables. Covariates included age; sex; race/ethnicity; education level; household income; US region; hyperlipidaemia; hypertension; chronic kidney disease; diabetes; baseline ASCVD; atrial fibrillation; use of LDL cholesterol-lowering therapy, anti-hypertension therapy and anti-platelet therapy; duration of use of LDL cholesterol-lowering therapy; Charlson Comorbidity Index; LDL cholesterol; total cholesterol and total triglycerides. CI, confidence interval; HR, hazard ratio; ASCVD, atherosclerotic cardiovascular disease

Including all individuals, higher lipoprotein(a) levels in clinically relevant categories were associated with continuously higher risk of a recurrent ASCVD event, in unadjusted analysis as well as in analyses adjusted for sex, race/ethnicity, and age or multivariable for all covariates (*Figure 3*). Compared to individuals with lipoprotein(a) < 15 nmol/L and, in analyses adjusted for sex, race/ethnicity, and age, the hazard ratio for recurrent ASCVD events was 1.04 (95% CI 1.01–1.07) for 15–79 nmol/L, 1.15 (1.12–1.19) for 80–179 nmol/L, 1.29 (1.25–1.33) for 180–299 nmol/L, and 1.45 (1.39–1.51) for individuals with lipoprotein(a) ≥ 300 nmol/L. When a second categorization of lipoprotein(a) levels of <75 nmol/L, 75–124 nmol/L, 125–199 nmol/L, 200–399 nmol/L, and ≥400 nmol/L was used based on guidance from the National Lipid Association,^2^ overall results of this sensitivity analysis were similar to those shown above (see Supplementary data online, *Table S8*).

**Figure 4:**
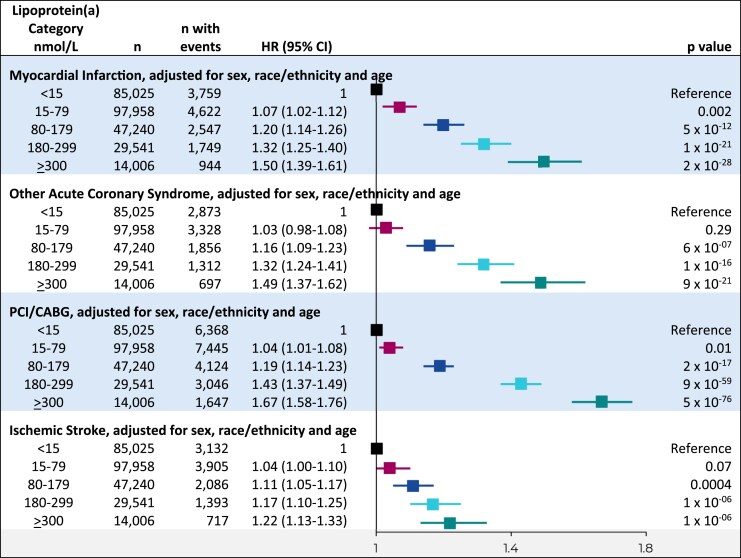
Risk of individual event components of recurrent ASCVD by clinically relevant categories of lipoprotein(a). Cox proportional hazards analyses adjusted for sex, race/ethnicity, and age with time to individual event component of recurrent ASCVD following initial ASCVD at baseline as the dependent variable and lipoprotein(a) categories as the independent variables. CI, confidence interval; HR, hazard ratio; ASCVD, atherosclerotic cardiovascular disease; PCI, percutaneous coronary intervention; CABG, coronary artery bypass surgery

These results were also similar for the individual components of ASCVD of myocardial infarction, other acute coronary syndrome, PCI/CABG, and ischaemic stroke, albeit with the lowest adjusted hazard ratios for ischaemic stroke (*Figure 4*).

### Lipoprotein(a) categories and recurrent atherosclerotic cardiovascular disease events by sex, race/ethnicity, baseline atherosclerotic cardiovascular disease, and baseline diabetes

**Figure 5:**
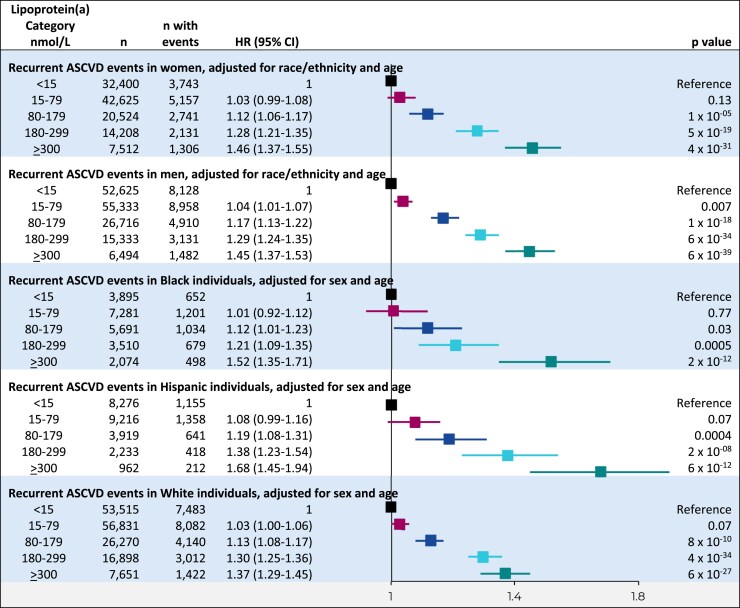
Risk of recurrent ASCVD events in lipoprotein(a) categories by sex and race/ethnicity. Cox proportional hazards analyses adjusted for sex, race/ethnicity, and age as appropriate with time to recurrent ASCVD event following initial ASCVD at baseline as the dependent variable and lipoprotein(a) categories as the independent variables. The interactions with lipoprotein(a) categories on risk of recurrent ASCVD event of race/ethnicity within the subgroup of women (*P* = .06); of race/ethnicity within the subgroup of men (*P* = .19); of sex within the subgroup of Black individuals (*P* = .74); of sex within the subgroup of Hispanic individuals (*P* = .84); and of sex within the subgroup of White individuals (*P* = .07) were formally tested. CI, confidence interval; HR, hazard ratio; ASCVD, atherosclerotic cardiovascular disease

Higher lipoprotein(a) levels in clinically relevant categories were associated with continuously increasing risk of a recurrent ASCVD event in each of the subgroups including women, men, and Black, Hispanic, or White individuals (*Figure 5*); however, at lipoprotein(a) ≥ 300 nmol/L, the magnitude of risk of a recurrent ASCVD event was slightly higher in Hispanic and Black than in White individuals.

Compared to Hispanic individuals with lipoprotein(a) < 15 nmol/L, the adjusted hazard ratios for recurrent ASCVD events were 1.08 (95% CI 0.99–1.16) for 15–79 nmol/L, 1.19 (1.08–1.31) for 80–179 nmol/L, 1.38 (1.23–1.54) for 200–299 nmol/L, and 1.68 (1.45–1.94) for individuals with lipoprotein(a) ≥ 300 nmol/L (*Figure 5*). Corresponding values were 1.01 (0.92–1.12), 1.12 (1.01–1.23), 1.21 (1.09–1.35), and 1.52 (1.35–1.71) in Black individuals, and 1.03 (1.00–1.06), 1.13 (1.08–1.17), 1.30 (1.25–1.36), and 1.37 (1.29–1.45) in White individuals, respectively.

**Figure 6:**
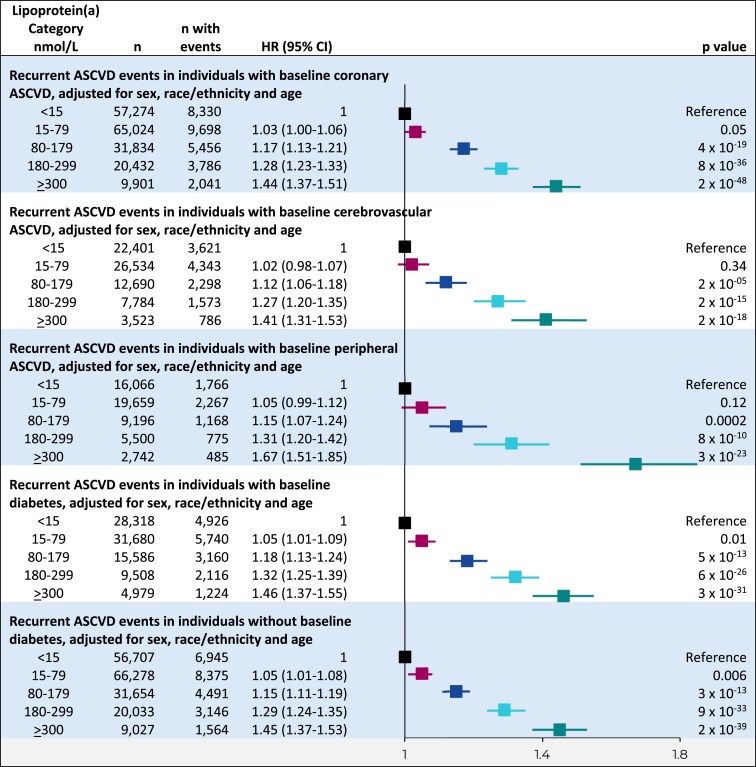
Risk of recurrent ASCVD events in lipoprotein(a) categories by baseline ASCVD and baseline diabetes. Cox proportional hazards analyses adjusted for sex, race/ethnicity, and age with time to recurrent ASCVD event following initial ASCVD at baseline as the dependent variable and lipoprotein(a) categories as the independent variables. Individuals were included in more than one ASCVD subgroup; 4009 individuals were not categorized into any baseline ASCVD subgroup due to lack of specificity of the ICD code. CI, confidence interval; HR, hazard ratio; ASCVD, atherosclerotic cardiovascular disease

Higher lipoprotein(a) levels were also associated with continuously increasing risk of a recurrent ASCVD event in the subgroup analyses by type of baseline ASCVD (coronary, cerebrovascular, peripheral) and diabetes status (yes/no; *Figure 6*).

Within the full ASCVD cohort, the interactions of sex (*P* = .61), race/ethnicity (*P* = .06), baseline ASCVD (*P* = .33), and baseline diabetes (*P* = .91) with lipoprotein(a) categories on risk of recurrent ASCVD event all had *P* > .05.

### Lipoprotein(a) categories and recurrent atherosclerotic cardiovascular disease events by LDL cholesterol-lowering therapy

Since there are no approved lipoprotein(a) lowering drugs, the current clinical strategy for treating individuals with high lipoprotein(a) is to ‘optimize’ other cardiovascular risk factors, most importantly by aggressive LDL cholesterol lowering.^1–4^ In an attempt to understand if such a strategy could work, we also conducted subgroup analyses by use of (i) high impact, (ii) low/moderate impact, and (iii) no LDL cholesterol-lowering therapy (*Table 1*).

**Figure 7:**
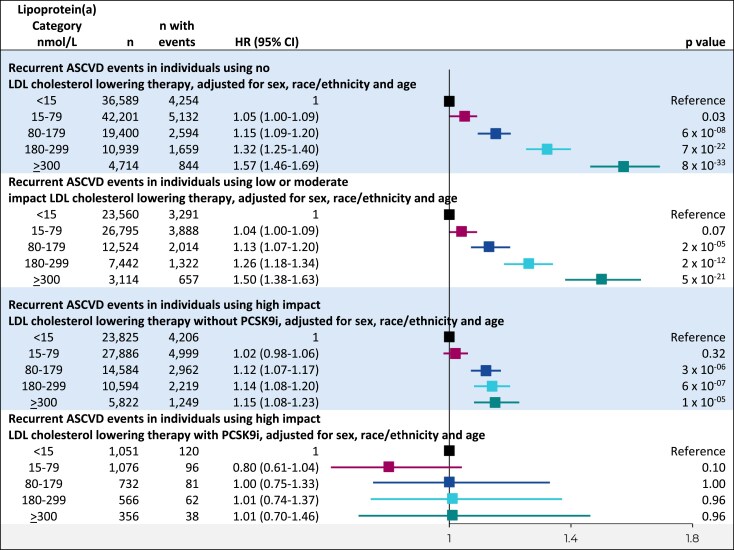
Risk of recurrent ASCVD events in lipoprotein(a) categories by impact of LDL cholesterol-lowering therapy. Cox proportional hazards analyses adjusted for sex, race/ethnicity, and age with time to recurrent ASCVD event following initial ASCVD at baseline as the dependent variable and lipoprotein(a) categories as the independent variables. CI, confidence interval; HR, hazard ratio; ASCVD, atherosclerotic cardiovascular disease; PCSK9i, proprotein convertase subtilisin/kexin type 9 inhibitor

The first analysis used a Cox proportional hazards model in subgroups defined by use of LDL cholesterol-lowering therapy in three classes (*Figure 7*). The use of high impact LDL cholesterol-lowering therapy vs no use or low/moderate impact LDL cholesterol-lowering therapy was associated with lower adjusted hazard ratios in those with lipoprotein(a) categories ≥ 180 nmol/L. The analyses that examined the interaction between LDL cholesterol-lowering therapy use and lipoprotein(a) categories had a *P* = 2 × 10^−8^. Within the subgroup of individuals using high impact LDL cholesterol-lowering therapy, persistent attenuation of lipoprotein(a)-associated risk at ≥180 nmol/L was observed in the group using no PCSK9i, and additional attenuation of lipoprotein(a)-associated risk in the group using PCSK9i (*Figure 7*) with associated test for interaction of *P* = .41.

**Figure 8:**
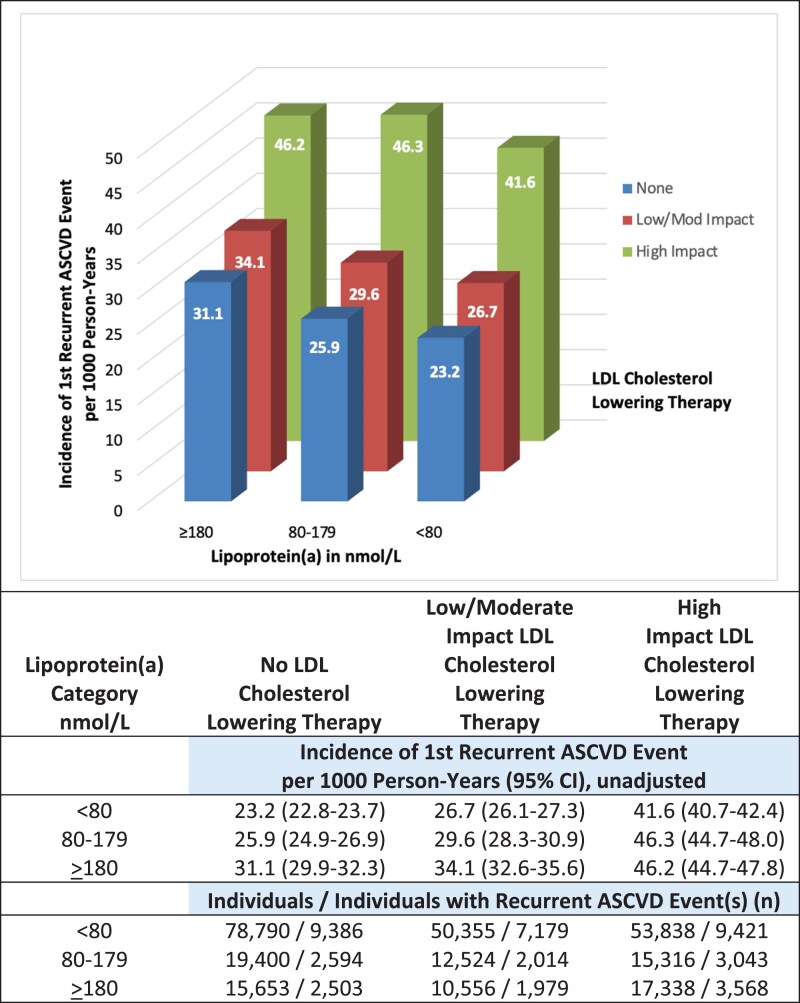
Absolute risk of recurrent ASCVD events in lipoprotein(a) categories by impact of LDL cholesterol-lowering therapy. Unadjusted Poisson regression analysis with recurrent ASCVD event following initial ASCVD at baseline as the dependent variable, categories of lipoprotein(a) and impact of LDL cholesterol-lowering therapy as independent variables, and log of the time in years to first recurrent ASCVD event, or end of follow-up if no recurrent ASCVD event, as the offset. CI, confidence interval; HR, hazard ratio; ASCVD, atherosclerotic cardiovascular disease

In an analysis using Poisson regression to count first recurrent ASCVD events per 1000 person-years, the association of lipoprotein(a) levels with events was assessed using a 3 × 3 categorization with lipoprotein(a) in three levels by use of LDL cholesterol-lowering therapy in three classes (*Figure 8*). Across individuals with lipoprotein(a) categories from <80, through 80–179, to ≥180 nmol/L, the rate of first recurrent ASCVD events per 1000 person-years was from 23.2 to 31.1 in those using no LDL cholesterol-lowering therapy, from 26.7 to 34.1 in those using low or moderate impact LDL cholesterol-lowering therapy, and from 41.6 to 46.2 in those using high impact LDL cholesterol-lowering therapy.

Although results from the above analysis (*Figures 7* and *8*) are consistent suggesting that the additional risk of recurrent ASCVD in those with lipoprotein(a) ≥ 180 nmol/L possibly is mitigated in individuals using high impact LDL cholesterol-lowering therapy, the second analysis (*Figure 8*) also shows that absolute risk of having a recurrent ASCVD event is lowest in individuals using no LDL cholesterol-lowering therapy and highest in individuals using high impact LDL cholesterol therapy. This seemingly counter-intuitive outcome is likely due to bias by indication for LDL cholesterol-lowering therapy and possibly by confounding, which is further discussed below.

## Discussion

Based on 273 770 US individuals with baseline ASCVD and available lipoprotein(a) levels, higher lipoprotein(a) levels were associated with continuously increasing risk of a recurrent ASCVD event regardless of sex, race/ethnicity (*Structured Graphical Abstract*), baseline ASCVD, and baseline diabetes. When examining risk of recurrent ASCVD in individuals with baseline ASCVD, our data illustrate that more women than men and more Black individuals than Hispanic and White individuals are at risk of a recurrent ASCVD event due to high lipoprotein(a). Additionally, our data show that use of high impact LDL cholesterol-lowering therapy possibly mitigates the deleterious effect of lipoprotein(a) ≥ 180 nmol/L, most pronounced in those receiving PCSK9i. These findings are novel.

The present study is both the largest by eight-fold and the most diverse in race/ethnicity to examine the relationship of higher lipoprotein(a) on recurrent ASCVD events in a contemporary secondary prevention setting. In support of our findings, in general population studies of mostly White/European individuals, the association of high lipoprotein(a) with increased cardiovascular events was documented by large observational studies and by Mendelian randomization studies of the UK Biobank (>460 000 individuals aged 40–69 years, 4% of whom had ASCVD at enrolment),^17–19^ and the Copenhagen General Population Study and Copenhagen City Heart Study (>120 000 individuals aged 20–100 years, 5% of whom had ASCVD at enrolment).^4,20–29^ However, in individuals with established ASCVD, the association of high lipoprotein(a) with recurrent ASCVD events has been less frequently assessed and analyses are hampered by limited power with inconsistent results.^30–34^

Results from our study differ from another cohort analysis of 16 419 individuals which concluded that cardiovascular risk plateaued beginning at lipoprotein(a) levels of ∼150–200 nmol/L in individuals with baseline ASCVD but did not plateau in individuals without baseline ASCVD.^32^ The large size of our study, including 273 770 ASCVD patients, relative to the other ASCVD studies allowed for comprehensive assessment of cardiovascular risk across the full continuum of lipoprotein(a) levels.^30–34^

Primary prevention studies have demonstrated that risk associated with high levels of lipoprotein(a) is similar in men and women, and that high lipoprotein(a) occurs more frequently in women beginning around 50 years of age corresponding with menopause and onwards.^11,35^ The current analysis confirms and extends those findings to women and men with baseline ASCVD by demonstrating very similar hazard ratios across the entire range of lipoprotein(a) categories in the two sexes and higher levels in women than men in those with baseline ASCVD.

In accordance with results in the primary prevention setting,^1^ our results in the secondary prevention setting show that lipoprotein(a) levels are higher in Black than in Hispanic and White individuals with ASCVD and that the risk of recurrent ASCVD is similar at higher lipoprotein(a) levels in individuals of different race/ethnicity. The totality of our data therefore illustrates that Black individuals with baseline ASCVD have higher risk of recurrent events due to higher lipoprotein(a) levels than Hispanic and White individuals.

In the present study, diabetes at baseline was observed in roughly a third of individuals in all lipoprotein(a) categories from <15 nmol/L through to ≥300 nmol/L, which at first sight could appear to be in contrast with a 38% higher risk of incident diabetes in the lowest vs highest lipoprotein(a) quintiles in the primary prevention setting.^1,4^ However, these two observations cannot be compared because the present study includes solely individuals with baseline ASCVD in a secondary prevention setting, and because we report diabetes at baseline and not incident diabetes. Results from the present study, showing that lipoprotein(a)-associated ASCVD risk is similar in individuals with and without baseline diabetes, also appear to contrast with results from a study in 27 756 individuals without ASCVD showing that lipoprotein(a)-associated ASCVD risk is elevated in individuals with diabetes.^35^ Additional research is needed to determine if the disparate results are due to differing populations (with vs without ASCVD), other design feature, or a spurious finding in the former study^35^ with less statistical power than in the present one including 273 770 individuals.

A major strength of the present analysis is that the data are current, expansive and based on the real-world experience of a diverse group of individuals with ASCVD from across the USA. It provides an alternative perspective to clinical trials and prospective registries with less diverse populations.

Limitations, naturally, also exist. First, this observational study used data from medical claims developed mainly for billing purposes. Mortality data are not reported, and there is potential for other data to be missing or inaccurate. Second, despite controlling for a wide array of covariates, some unmeasured covariates (such as obesity, family history and smoking, which are all sparsely and poorly coded in claims data) may introduce bias into the analysis. That said, because levels of lipoprotein(a) are >90% genetically determined,^4^ confounding is unlikely to have a major influence on the main results according to the principle of Mendelian randomization.^9,10^ Third, individuals with lipoprotein(a) results available may be different from those who have never undergone an lipoprotein(a) assessment. Fourth, although this analysis included only nmol/L measures, and most measures were derived using a highly standardized process, a small fraction of lipoprotein measures may have used non-standard methods. Fifth, although all comparisons were made within a cohort of individuals with the same entry criteria and flow through pre-index and post-index elements of the study, there may be some immortal time bias introduced into this analysis by including individuals who entered the database with ASCVD and others who developed ASCVD after entering the database. Similarly, index event bias is introduced by including only individuals with ASCVD; however, this is exactly what happens in many randomized trials focusing on secondary prevention of ASCVD. In addition, the possibility of some immortal time bias and/or index event bias is no different from that of any other relevant epidemiological study.

Finally, limitations are particularly important to consider in the analyses assessing use of (i) high impact, (ii) low/moderate impact, and (iii) no LDL cholesterol-lowering therapy, because such medication use is largely not genetically determined. Such observational data are therefore prone to confounding and bias, particularly when it comes to medication use in the form of bias by indication. This means that those at the highest risk of recurrent ASCVD as judged by their physician are the ones most likely to use high impact vs low/moderate impact or no LDL cholesterol-lowering therapy. This is illustrated in *Figure 8*, where those using high impact LDL cholesterol-lowering therapy have the highest absolute risk of a first recurrent ASCVD event, irrespective of their lipoprotein(a) levels. Nevertheless, the present result cannot exclude that high impact LDL cholesterol-lowering therapy possibly mitigates a deleterious effect of lipoprotein(a) ≥ 180 nmol/L with respect to recurrent ASCVD. That said, this hypothesis can only be finally documented in a randomized controlled trial, a trial which is unlikely to be conducted as three cardiovascular endpoint trials using aggressive lipoprotein(a)-lowering therapies are ongoing.^1^

## Conclusion

Based on 273 770 US individuals with baseline ASCVD, higher lipoprotein(a) levels were associated with continuously increasing risk of a recurrent ASCVD event regardless of sex and race/ethnicity. Furthermore, our data show that for the same level of lipoprotein(a), more women than men and more Black individuals than Hispanic and White individuals are at risk of a recurrent ASCVD event due to high lipoprotein(a). Lipoprotein(a)-associated risk was similar regardless of baseline ASCVD and diabetes, and may have been partially mitigated by the use of high impact LDL cholesterol-lowering therapy, most pronounced in the case of PCSK9i use.

These data point at an unmet medical need for lipoprotein(a)-lowering treatment in the entire US racially and ethnically diverse ASCVD population. They also highlight the need to measure lipoprotein(a) and consider the magnitude of higher levels when assessing the risk of recurrent ASCVD events in all individuals with ASCVD. Should the ongoing trials of lipoprotein(a)-lowering agents prove safe and effective, these agents should be added to the armamentarium of effective lipid-altering medications.

## Supplementary Material

ehaf297_Supplementary_Data

## Declarations

### Data Availability

The data underlying this article were originally acquired from Symphony Health an ICON plc Company (Blue Bell, PA, USA). The derived data generated in this research will be shared on reasonable request to the corresponding author.

### Pre-registered Clinical Trial Number

None supplied.

## References

[1] Nordestgaard BG, Langsted A. Lipoprotein(a) and cardiovascular disease. Lancet 2024;404:1255–64. 10.1016/S0140-6736(24)01308-4 39278229

[2] Koschinsky ML, Bajaj A, Boffa MB, Dixon DL, Ferdinand KC, Gidding SS, et al A focused update to the 2019 NLA scientific statement on use of lipoprotein(a) in clinical practice. J Clin Lipidol 2024;18:e308–19. 10.1016/j.jacl.2024.03.001 38565461

[3] Reyes-Soffer G, Ginsberg HN, Berglund L, Duell PB, Heffron SP, Kamstrup PR, et al Lipoprotein(a): a genetically determined, causal, and prevalent risk factor for atherosclerotic cardiovascular disease. A scientific statement from the American Heart Association. Arterioscler Thromb Vasc Biol 2022;42:e48–60. 10.1161/ATV.0000000000000147 34647487 PMC9989949

[4] Kronenberg F, Mora S, Stroes ESG, Ference BA, Arsenault BJ, Berglund L, et al Lipoprotein(a) in atherosclerotic cardiovascular disease and aortic stenosis: a European Atherosclerosis Society consensus statement. Eur Heart J 2022;43:3925–46. 10.1093/eurheartj/ehac361 36036785 PMC9639807

[5] Nordestgaard BG, Langsted A. Lipoprotein (a) as a cause of cardiovascular disease: insights from epidemiology, genetics, and biology. J Lipid Res 2016;57:1953–75. 10.1194/jlr.R071233 27677946 PMC5087876

[6] Tsimikas S, Karwatowska-Prokopczuk E, Gouni-Berthold I, Tardif J-C, Baum SJ, Steinhagen-Thiessen E, et al Lipoprotein(a) reduction in persons with cardiovascular disease. N Engl J Med 2020;382:244–55. 10.1056/NEJMoa1905239 31893580

[7] O'Donoghue ML, Rosenson RS, Gencer B, López JAG, Lepor NE, Baum SJ, et al Small interfering RNA to reduce lipoprotein(a) in cardiovascular disease. N Engl J Med 2022;387:1855–64. 10.1056/NEJMoa2211023 36342163

[8] Nissen SE, Wolski K, Balog C, Swerdlow DI, Scrimgeour AC, Rambaran C, et al Single ascending dose study of a short interfering RNA targeting lipoprotein(a) production in individuals with elevated plasma lipoprotein(a) levels. JAMA 2022;327:1679–87. 10.1001/jama.2022.5050 35368052 PMC8978050

[9] Smith GD, Ebrahim S. Mendelian randomization: prospects, potentials, and limitations. Int J Epidemiol 2004;33:30–42. 10.1093/ije/dyh132 15075143

[10] Benn M, Nordestgaard BG. From genome-wide association studies to Mendelian randomization: novel opportunities for understanding cardiovascular disease causality, pathogenesis, prevention, and treatment. Cardiovasc Res 2018;114:1192–208. 10.1093/cvr/cvy045 29471399

[11] Simony SB, Mortensen MB, Langsted A, Afzal S, Kamstrup PR, Nordestgaard BG. Sex differences of lipoprotein(a) levels and associated risk of morbidity and mortality by age: the Copenhagen General Population Study. Atherosclerosis 2022; 355:7682. 10.1016/j.atherosclerosis.2022.06.1023 35803767

[12] Cuchel M, Lee PC, Hudgins LC, Duell PB, Ahmad Z, Baum SJ, et al Contemproary homozygous familial hypercholesterolemia in the United States: insights form the CASCADE FH Registry. J Am Heart Assoc 2023;12:e029175. 10.1161/JAHA.122.029175 37119068 PMC10227232

[13] Tsai T-Y, Lin J-F, Tu Y-K, Lee J-H, Hsiao Y-T, Sung S-F, et al Validation of ICD-10-CM diagnostic codes for identifying patients with ST-elevation and non-ST-elevation myocardial infarction in a National Health Insurance Claims database. Clin Epidemiol 2023;15:1027–39. 10.2147/CLEP.S431231 37868152 PMC10590151

[14] Strom JB, Zhao Y, Faridi KF, Tamez H, Butala NM, Valsdottir LR, et al Comparison of clinical trials and administrative claims to identify stroke among patients undergoing aortic valve replacement: findings from the extending trial-based evaluations of medical therapies using novel sources of data (EXTEND) study. Circ Cardiovasc Interv 2019;12:e008231. 10.1161/CIRCINTERVENTIONS.119.008231 31694411 PMC7212938

[15] Quan H, Sundararajan V, Halfon P, Fong A, Burnand B, Luthi J-C, et al Coding algorithms for defining comorbidities in ICD-9-CM and ICD-10 administrative data. Med Care 2005;43:1130–9. 10.1097/01.mlr.0000182534.19832.83 16224307

[16] Croxford R . Restricted Cubic Spline Regression: A Brief Introduction. Toronto, ON: Institute for Clinical Evaluative Sciences, 2016. https://support.sas.com/resources/papers/proceedings16/5621-2016.pdf (16 August 2024, date last accessed).

[17] Patel AP, Wang M, Pirruccello JP, Ellinor PT, Ng K, Kathiresan S, et al Lp(a) (lipoprotein(a)) concentrations and incident atherosclerotic cardiovascular disease. Arterioscler Thromb Vasc Biol 2021;41:465–74. 10.1161/ATVBAHA.120.315291 33115266 PMC7769893

[18] Trinder M, Uddin MM, Finneran P, Aragam KG, Natarajan P. Clinical utility of lipoprotein(a) and LPA genetic risk score in risk prediction of incident atherosclerotic cardiovascular disease. JAMA Cardiol 2021;6:287–95. 10.1001/jamacardio.2020.5398 33021622 PMC7539232

[19] Emdin CA, Khera AV, Natarajan P, Klarin D, Won H-H, Peloso GM, et al Phenotypic characterization of genetically lowered human lipoprotein(a) levels. J Am Coll Cardiol 2016;68:2761–72. 10.1016/j.jacc.2016.10.033 28007139 PMC5328146

[20] Kamstrup PR, Benn M, Tybjaerg-Hansen A, Nordestgaard BG. Extreme lipoprotein(a) levels and risk of myocardial infarction in the general population: the Copenhagen City Heart Study. Circulation 2008;117:176–84. 10.1161/CirculationAHA.107.715698 18086931

[21] Kamstrup PR, Tybjaerg-Hansen A, Steffensen R, Nordestgaard BG. Genetically elevated lipoprotein(a) and increased risk of myocardial infarction. JAMA 2009;301:2331–9. 10.1001/jama.2009.801 19509380

[22] Nordestgaard BG, Chapman MJ, Ray K, Borén J, Andreotti F, Watts GF, et al Lipoprotein(a) as a cardiovascular risk factor: current status. Eur Heart J 2010;31:2844–53. 10.1093/eurheartj/ehq386 20965889 PMC3295201

[23] Thanassoulis G, Campbell CY, Owens DS, Smith JG, Smith AV, Peloso GM, et al Genetic associations with valvular calcification and aortic stenosis. N Engl J Med 2013;368:503–12. 10.1056/NEJMoa1109034 23388002 PMC3766627

[24] Kamstrup PR, Tybjaerg-Hansen A, Nordestgaard BG. Elevated lipoprotein(a) and risk of aortic valve stenosis in the general population. J Am Coll Cardiol 2014;63:470–7. 10.1016/j.jacc.2013.09.038 24161338

[25] Kamstrup PR, Nordestgaard BG. Elevated lipoprotein(a) levels, LPA risk genotypes, and increased risk of heart failure in the general population. JACC Heart Fail 2016;4:78–87. 10.1016/j.jchf.2015.08.006 26656145

[26] Langsted A, Kamstrup PR, Nordestgaard BG. High lipoprotein(a) and high risk of mortality. Eur Heart J 2019;40:2760–70. 10.1093/eurheartj/ehy902 30608559

[27] Langsted A, Nordestgaard BG, Kamstrup PR. Elevated lipoprotein(a) and risk of ischemic stroke. J Am Coll Cardiol 2019;74:54–66. 10.1016/j.jacc.2019.03.524 31272552

[28] Langsted A, Nordestgaard BG, Kamstrup PR. Low lipoprotein(a) levels and risk of disease in a large, contemporary, general population study. Eur Heart J 2021;42:1147–56. 10.1093/eurheartj/ehaa1085 33724357

[29] Thomas PE, Vedel-Krogh S, Nielsen SF, Nordestgaard BG, Kamstrup PR. Lipoprotein(a) and risks of peripheral artery disease, abdominal aortic aneurysm, and major adverse limb events. J Am Coll Cardiol 2023;82:2265–76. 10.1016/j.jacc.2023.10.009 38057068

[30] Albers JJ, Slee A, O'Brien KD, Robinson JG, Kashyap ML, Kwiterovich J, et al Relationship of apolipoproteins A-1 and B, and lipoprotein(a) to cardiovascular outcomes: the AIM-HIGH trial (Atherothrombosis Intervention in Metabolic Syndrome with Low HDL/High Triglyceride and Impact on Global Health Outcomes). J Am Coll Cardiol 2013;62:1575–9. 10.1016/j.jacc.2013.06.051 23973688 PMC3800510

[31] Madsen CM, Kamstrup PR, Langsted A, Varbo A, Nordestgaard BG. Lipoprotein(a)-lowering by 50 mg/dL (105 nmol/L) may be needed to reduce cardiovascular disease 20% in secondary prevention. A population-based study. Arterioscler Thromb Vasc Biol 2020;40:255–66. 10.1161/ATVBAHA.119.312951 31578080

[32] Berman AN, Biery DW, Besser SA, Singh A, Shiyovich A, Weber BN, et al Lipoprotein(a) and major adverse cardiovascular events in patients with or without baseline atherosclerotic cardiovascular disease. JACC 2024;83:873–86. 10.1016/j.jacc.2023.12.031 38418000 PMC12161880

[33] Welsh P, Al Zabiby A, Byrne H, Benbow HR, Itani T, Farries G, et al Elevated lipoprotein(a) increases risk of subsequent major adverse cardiovascular events (MACE) and coronary revascularization in incident ASCVD patients: a cohort study from the UK biobank. Atherosclerosis 2024; 389:117437. 10.1016/j.atherosclerosis.2023.117437 38219651

[34] Yuan S, Li F, Zhang H, Zeng J, Su X, Qu J, et al Impact of high lipoprotein(a) on long-term survival following coronary artery bypass grafting. J Am Heart Assoc 2024;13:e031322. 10.1161/JAHA.123.031322 38240214 PMC11056181

[35] Wong ND, Fan W, Hu X, Ballantyne C, Hoodgeveen RC, Tsai MY, et al Lipoprotein(a) and long-term cardiovascular risk in a multi-ethnic pooled prospective cohort. J Am Coll Cardiol 2024;83:1511–25. 10.1016/j.jacc.2024.02.031 38631771

