# Supplementary material for: Lipoprotein(a) and recurrent atherosclerotic cardiovascular events: the US Family Heart Database
Source: Eur Heart J. 2025 May 7;46(44):4762–75. doi: 10.1093/eurheartj/ehaf297 (PMC12634116; doi:10.1093/eurheartj/ehaf297)
Supplement: ehaf297_Supplementary_Data [file ehaf297_supplementary_data.zip › supp_table1.pdf]

Table S1a. Baseline ASCVD: Clinical conditions with associated ICD codes

ASCVD = atherosclerotic cardiovascular disease; CABG = coronary artery bypass graft; MI = myocardial infarction; PCI = percutaneous coronary intervention; PVD = peripheral vascular disease; TIA = transient ischemic attack; Hx = history.

Clinical condition	ICD code for clinical condition
**ASCVD**	33335, 411, 411.0, 411.8, 411.89, 414, 414.00, 414.01, 414.2, 414.3, 414.4, 414.8, 414.9, 429.2, 440, 440.0, 440.8, 440.9, 441.3, 441.4, 444.0, 444.1, 92974, I24.1, I25.10, I25.5, I25.6, I25.8, I25.82, I25.83, I25.84, I25.89, I25.9, I70.0, I70.8, I70.90, I70.91
**ASCVD of CABG**	414.02, 414.03, 414.04, 414.05, I25.71, I25.72, I25.73, I25.79, I25.810
**ASCVD of CABG/Angina**	I25.70, I25.702, I25.708, I25.709, I25.711, I25.712, I25.718, I25.719, I25.721, I25.722, I25.728, I25.729, I25.731, I25.732, I25.738, I25.739, I25.768, I25.769, I25.791, I25.792, I25.798, I25.799
**ASCVD of CABG/Angina/Transplant**	I25.761
**ASCVD of CABG/ Transplant**	I25.7, I25.76, I25.812
**ASCVD of CABG/Transplant/Angina**	I25.762
**ASCVD of CABG/Transplant/ Unstable Angina**	I25.760
**ASCVD of CABG/ Unstable Angina**	I25.700, I25.701, I25.710, I25.720, I25.730, I25.790
**ASCVD/Angina**	I25.11, I25.111, I25.112, I25.118, I25.119, I25.751, I25.758, I25.759
**ASCVD/Hx of CABG**	33530, V45.81, Z95.1
**ASCVD/Hx of CABG/PCI**	Z95.5
**ASCVD/Hx of MI**	410.02, 410.12, 410.22, 410.32, 410.42, 410.52, 410.62, 410.72, 410.82, 410.92, 412, I23, I23.0, I23.1, I23.2, I23.3, I23.4, I23.5, I23.6, I23.8, I25.2
**ASCVD/Transplant**	414.06, I25.75, I25.811

Clinical condition	ICD code for clinical condition
**ASCVD/Transplant/ Angina**	I25.752
**ASCVD/Transplant/ Unstable Angina**	I25.750
**ASCVD/Unstable Angina**	I25.110
**Acute Ischemic Heart Disease**	02C00Z6, 02C00ZZ, 02C03Z6, 02C03ZZ, 02C04Z6, 02C04ZZ, 02C10Z6, 02C10ZZ, 02C13Z6, 02C13ZZ, 02C14Z6, 02C14ZZ, 02C20Z6, 02C20ZZ, 02C23Z6, 02C23ZZ, 02C24Z6, 02C24ZZ, 02C30Z6, 02C30ZZ, 02C33Z6, 02C33ZZ, 02C34Z6, 02C34ZZ, 33140, 33141, 3604, 3609, 36593, 3E07017, 3E070PZ, 3E07317, 3E073PZ, 411.81, 92973, 92975, 92977, C1714, C1724, C1725, C9603, I24.0, I24.8, I24.9, X2C0361, X2C1361, X2C2361, X2C3361
**Angina**	413, 413.0, 413.1, 413.9, I20, I20.1, I20.2, I20.8, I20.89, I20.9, I23.7
**TIA**	435, 435.8, 435.9, G45.3, G45.8, G45.9
**TIA/Cerebrovascular disease**	V12.54, Z86.73
**Unstable Angina**	I20.0

Clinical condition	ICD code for clinical condition
**CABG**	0210083, 0210088, 0210089, 021008C, 021008F, 021008W, 0210093, 0210098, 0210099, 021009C, 021009F, 021009W, 02100A3, 02100A8, 02100A9, 02100AC, 02100AF, 02100AW, 02100J3, 02100J8, 02100J9, 02100JC, 02100JF, 02100JW, 02100K3, 02100K8, 02100K9, 02100KC, 02100KF, 02100KW, 02100Z3, 02100Z8, 02100Z9, 02100ZC, 02100ZF, 0210344, 02103D4, 0210444, 0210483, 0210488, 0210489, 021048C, 021048F, 021048W, 0210493, 0210498, 0210499, 021049C, 021049F, 021049W, 02104A3, 02104A8, 02104A9, 02104AC, 02104AF, 02104AW, 02104D4, 02104J3, 02104J8, 02104J9, 02104JC, 02104JF, 02104JW, 02104K3, 02104K8, 02104K9, 02104KC, 02104KF, 02104KW, 02104Z3, 02104Z8, 02104Z9, 02104ZC, 02104ZF, 0211083, 0211088, 0211089, 021108C, 021108F, 021108W, 0211093, 0211098, 0211099, 021109C, 021109F, 021109W, 02110A3, 02110A8, 02110A9, 02110AC, 02110AF, 02110AW, 02110J3, 02110J8, 02110J9, 02110JC, 02110JF, 02110JW, 02110K3, 02110K8, 02110K9, 02110KC, 02110KF, 02110KW, 02110Z3, 02110Z8, 02110Z9, 02110ZC, 02110ZF, 0211344, 02113D4, 0211444, 0211483, 0211488, 0211489, 021148C, 021148F, 021148W, 0211493, 0211498, 0211499, 021149C, 021149F, 021149W, 02114A3, 02114A8, 02114A9, 02114AC, 02114AF, 02114AW, 02114D4, 02114J3, 02114J8, 02114J9, 02114JC, 02114JF, 02114JW, 02114K3, 02114K8, 02114K9, 02114KC, 02114KF, 02114KW, 02114Z3, 02114Z8, 02114Z9, 02114ZC, 02114ZF, 0212083, 0212088, 0212089, 021208C, 021208F, 021208W, 0212093, 0212098, 0212099, 021209C, 021209F, 021209W, 02120A3, 02120A8, 02120A9, 02120AC, 02120AF, 02120AW, 02120J3, 02120J8, 02120J9, 02120JC, 02120JF, 02120JW, 02120K3, 02120K8, 02120K9, 02120KC, 02120KF, 02120KW, 02120Z3, 02120Z8, 02120Z9, 02120ZC, 02120ZF, 0212344, 02123D4, 0212444, 0212483, 0212488, 0212489, 021248C, 021248F, 021248W, 0212493, 0212498, 0212499, 021249C, 021249F, 021249W, 02124A3, 02124A8, 02124A9, 02124AC, 02124AF, 02124AW, 02124D4, 02124J3, 02124J8, 02124J9, 02124JC, 02124JF, 02124JW, 02124K3, 02124K8, 02124K9, 02124KC, 02124KF, 02124KW, 02124Z3, 02124Z8, 02124Z9, 02124ZC, 02124ZF, 0213083, 0213088, 0213089, 021308C, 021308F, 021308W, 0213093, 0213098, 0213099, 021309C, 021309F, 021309W, 02130A3, 02130A8, 02130A9, 02130AC, 02130AF, 02130AW, 02130J3, 02130J8, 02130J9, 02130JC, 02130JF, 02130JW, 02130K3, 02130K8, 02130K9, 02130KC, 02130KF, 02130KW, 02130Z3, 02130Z8, 02130Z9, 02130ZC, 02130ZF, 0213344, 02133D4, 0213444, 0213483, 0213488, 0213489, 021348C, 021348F, 021348W, 0213493, 0213498, 0213499, 021349C, 021349F, 021349W, 02134A3, 02134A8, 02134A9, 02134AC, 02134AF, 02134AW, 02134D4, 02134J3, 02134J8, 02134J9, 02134JC, 02134JF, 02134JW, 02134K3, 02134K8, 02134K9, 02134KC, 02134KF, 02134KW, 02134Z3, 02134Z8, 02134Z9, 02134ZC, 02134ZF, 33315, 33508, 33510, 33511, 33512, 33513, 33514, 33516, 33517, 33518, 33519, 33521, 33522, 33523, 33533, 33534, 33535, 33536, 33572, 33860, 33863, 33864, 33870, 36.1, 36.10, 36.11, 36.12, 36.13, 36.14, 36.15, 36.16, 36.17, 36.18, 36.19, 36.2, 3610, 3611, 3612, 3613, 3614, 3615, 3616, 3619, S2205, S2206

Clinical condition	ICD code for clinical condition
**Cerebrovascular disease**	031H09G, 031H0AG, 031H0JG, 031H0KG, 031H0ZG, 031J09G, 031J0AG, 031J0JG, 031J0KG, 031J0ZG, 031S09G, 031S0AG, 031S0JG, 031S0KG, 031S0ZG, 031T09G, 031T0AG, 031T0JG, 031T0KG, 031T0ZG, 037G34Z, 037G3DZ, 037G3ZZ, 037G44Z, 037G4DZ, 037G4ZZ, 037H34Z, 037H3DZ, 037H3ZZ, 037H44Z, 037H4DZ, 037H4ZZ, 037J34Z, 037J3DZ, 037J3ZZ, 037J44Z, 037J4DZ, 037J4ZZ, 037K34Z, 037K3DZ, 037K3ZZ, 037K44Z, 037K4DZ, 037K4ZZ, 037L34Z, 037L3DZ, 037L3ZZ, 037L44Z, 037L4DZ, 037L4ZZ, 037M34Z, 037M3DZ, 037M3ZZ, 037M44Z, 037M4DZ, 037M4ZZ, 037N34Z, 037N3DZ, 037N3ZZ, 037N44Z, 037N4DZ, 037N4ZZ, 03CG3Z6, 03CG3ZZ, 03CH3ZZ, 03CJ3ZZ, 03CK3ZZ, 03CL3ZZ, 03CM3ZZ, 03CN3ZZ, 35301, 35501, 35506, 35508, 35509, 35510, 35601, 35606, 35642, 411.1, 433, 433.0, 433.00, 433.1, 433.10, 433.2, 433.20, 433.3, 433.30, 433.8, 433.80, 433.9, 433.90, 434, 434.0, 434.00, 434.9, 436, 437, 437.0, 437.1, 437.8, 437.9, 438.0, 438.10, 438.11, 438.12, 438.13, 438.14, 438.19, 438.20, 438.21, 438.22, 438.30, 438.31, 438.32, 438.40, 438.41, 438.42, 438.50, 438.51, 438.52, 438.53, 438.6, 438.7, 438.8, 438.81, 438.82, 438.83, 438.84, 438.85, 438.89, 438.9, 61630, 61635, G45.1, G45.2, G46.0, G46.1, G46.2, G46.3, G46.4, G46.5, G46.6, G46.7, G46.8, I67.2, I67.81, I67.82, I67.848, I67.89, I67.9
**Ischemic Stroke**	433.01, 433.11, 433.21, 433.31, 433.41, 433.51, 433.61, 433.71, 433.81, 433.91, 434.01, 434.11, 434.91, I63, I63.0, I63.00, I63.01, I63.011, I63.012, I63.013, I63.019, I63.02, I63.03, I63.031, I63.032, I63.033, I63.039, I63.09, I63.1, I63.10, I63.111, I63.112, I63.113, I63.119, I63.12, I63.131, I63.132, I63.133, I63.139, I63.19, I63.2, I63.20, I63.21, I63.211, I63.212, I63.213, I63.219, I63.22, I63.23, I63.231, I63.232, I63.233, I63.239, I63.29, I63.3, I63.30, I63.31, I63.311, I63.312, I63.313, I63.319, I63.32, I63.321, I63.322, I63.323, I63.329, I63.33, I63.331, I63.332, I63.333, I63.339, I63.34, I63.341, I63.342, I63.343, I63.349, I63.39, I63.40, I63.411, I63.412, I63.413, I63.419, I63.421, I63.422, I63.423, I63.429, I63.431, I63.432, I63.433, I63.439, I63.441, I63.442, I63.443, I63.449, I63.49, I63.5, I63.50, I63.51, I63.511, I63.512, I63.513, I63.519, I63.52, I63.521, I63.522, I63.523, I63.529, I63.53, I63.531, I63.532, I63.533, I63.539, I63.54, I63.541, I63.542, I63.543, I63.549, I63.59, I63.6, I63.8, I63.81, I63.89, I63.9
**MI**	410, 410.0, 410.00, 410.01, 410.1, 410.10, 410.11, 410.2, 410.20, 410.21, 410.3, 410.30, 410.31, 410.4, 410.40, 410.41, 410.5, 410.50, 410.51, 410.6, 410.60, 410.61, 410.7, 410.70, 410.71, 410.8, 410.80, 410.81, 410.9, 410.90, 410.91, I21, I21.0, I21.01, I21.02, I21.09, I21.1, I21.11, I21.19, I21.2, I21.21, I21.29, I21.3, I21.4, I21.9, I21.A1, I21.A9, I22, I22.0, I22.1, I22.2, I22.8, I22.9

Clinical condition	ICD code for clinical condition
**PCI**	0.66, 00.45, 00.46, 00.47, 00.48, 0270046, 027004Z, 0270056, 027005Z, 0270066, 027006Z, 0270076, 027007Z, 02700D6, 02700DZ, 02700EZ, 02700F6, 02700FZ, 02700G6, 02700GZ, 02700T6, 02700TZ, 02700Z6, 02700ZZ, 0270346, 027034Z, 0270356, 027035Z, 0270366, 027036Z, 0270376, 027037Z, 02703D6, 02703DZ, 02703E6, 02703EZ, 02703F6, 02703FZ, 02703G6, 02703GZ, 02703T6, 02703TZ, 02703Z6, 02703ZZ, 0270446, 027044Z, 0270456, 027045Z, 0270466, 027046Z, 0270476, 027047Z, 02704D6, 02704DZ, 02704E6, 02704EZ, 02704F6, 02704FZ, 02704G6, 02704GZ, 02704T6, 02704TZ, 02704Z6, 02704ZZ, 0271046, 027104Z, 0271056, 027105Z, 0271066, 027106Z, 0271076, 027107Z, 02710D6, 02710DZ, 02710EZ, 02710F6, 02710FZ, 02710G6, 02710GZ, 02710T6, 02710TZ, 02710Z6, 02710ZZ, 0271346, 027134Z, 0271356, 027135Z, 0271366, 027136Z, 0271376, 027137Z, 02713D6, 02713DZ, 02713E6, 02713EZ, 02713F6, 02713FZ, 02713G6, 02713GZ, 02713T6, 02713TZ, 02713Z6, 02713ZZ, 0271446, 027144Z, 0271456, 027145Z, 0271466, 027146Z, 0271476, 027147Z, 02714D6, 02714DZ, 02714E6, 02714EZ, 02714F6, 02714FZ, 02714G6, 02714GZ, 02714T6, 02714TZ, 02714Z6, 02714ZZ, 0272046, 027204Z, 0272056, 027205Z, 0272066, 027206Z, 0272076, 027207Z, 02720D6, 02720DZ, 02720EZ, 02720F6, 02720FZ, 02720G6, 02720GZ, 02720T6, 02720TZ, 02720Z6, 02720ZZ, 0272346, 027234Z, 0272356, 027235Z, 0272366, 027236Z, 0272376, 027237Z, 02723D6, 02723DZ, 02723E6, 02723EZ, 02723F6, 02723FZ, 02723G6, 02723GZ, 02723T6, 02723TZ, 02723Z6, 02723ZZ, 0272446, 027244Z, 0272456, 027245Z, 0272466, 027246Z, 0272476, 027247Z, 02724D6, 02724DZ, 02724E6, 02724EZ, 02724F6, 02724FZ, 02724G6, 02724GZ, 02724T6, 02724TZ, 02724Z6, 02724ZZ, 0273046, 027304Z, 0273056, 027305Z, 0273066, 027306Z, 0273076, 027307Z, 02730D6, 02730DZ, 02730EZ, 02730F6, 02730FZ, 02730G6, 02730GZ, 02730T6, 02730TZ, 02730Z6, 02730ZZ, 0273346, 027334Z, 0273356, 027335Z, 0273366, 027336Z, 0273376, 027337Z, 02733D6, 02733DZ, 02733E6, 02733EZ, 02733F6, 02733FZ, 02733G6, 02733GZ, 02733T6, 02733TZ, 02733Z6, 02733ZZ, 0273446, 027344Z, 0273456, 027345Z, 0273466, 027346Z, 0273476, 027347Z, 02734D6, 02734DZ, 02734E6, 02734EZ, 02734F6, 02734FZ, 02734G6, 02734GZ, 02734T6, 02734TZ, 02734Z6, 02734ZZ, 02H00DZ, 02H00YZ, 02H03DZ, 02H03YZ, 02H04DZ, 02H04YZ, 02H10DZ, 02H10YZ, 02H13DZ, 02H13YZ, 02H14DZ, 02H14YZ, 02H20DZ, 02H20YZ, 02H23DZ, 02H23YZ, 02H24DZ, 02H24YZ, 02H30DZ, 02H30YZ, 02H33DZ, 02H33YZ, 02H34DZ, 02H34YZ, 36.0, 36.00, 36.01, 36.02, 36.03, 36.04, 36.05, 36.06, 36.07, 36.08, 36.09, 3603, 3606, 3607, 3633, 3634, 37205, 37206, 37207, 37208, 92920, 92921, 92924, 92925, 92928, 92929, 92933, 92934, 92980, 92981, 92982, 92984, 92995, 92996, 92997, 92998, C1874, C1875, C1876, C1877, C1885, C2623, C9600, C9601, C9602, C9604, C9605, C9606, C9607, C9608, G0290, G0291
**PCI/CABG**	021K4Z8, 36.3, 36.30, 36.31, 36.32, 36.33, 36.34, 36.35, 36.36, 36.37, 36.38, 36.39, 3631, 3632, 3639, 92937, 92938, 92941, 92943, 92944, Z95.818, Z95.9
**PVD/ASCVD**	444.8, 444.89, 444.9, 445

Clinical condition	ICD code for clinical condition
**PVD**	0312090, 0312091, 0312093, 0312094, 0312096, 0312097, 0312098, 0312099, 031209B, 031209C, 031209D, 031209J, 031209K, 03120A1, 03120A4, 03120A6, 03120A7, 03120A8, 03120A9, 03120AB, 03120AC, 03120AD, 03120AF, 03120AJ, 03120AK, 03120J0, 03120J1, 03120J3, 03120J4, 03120J6, 03120J7, 03120J8, 03120J9, 03120JB, 03120JC, 03120JD, 03120JF, 03120JJ, 03120JK, 03120JW, 03120K1, 03120K6, 03120K7, 03120K8, 03120K9, 03120KB, 03120KC, 03120KD, 03120KF, 03120KK, 03120Z0, 03120Z1, 03120Z6, 03120Z7, 03120Z8, 03120Z9, 03120ZB, 03120ZC, 03120ZD, 03120ZF, 03120ZJ, 03120ZK, 0313090, 0313093, 0313096, 0313097, 0313098, 0313099, 031309B, 031309C, 031309D, 031309F, 031309J, 031309W, 03130A0, 03130A1, 03130A6, 03130A7, 03130A8, 03130A9, 03130AB, 03130AC, 03130AD, 03130AJ, 03130AK, 03130AW, 03130J0, 03130J1, 03130J3, 03130J6, 03130J7, 03130J8, 03130J9, 03130JB, 03130JC, 03130JD, 03130JJ, 03130JK, 03130JM, 03130JW, 03130K0, 03130K1, 03130K6, 03130K7, 03130K8, 03130K9, 03130KB, 03130KC, 03130KD, 03130KJ, 03130Z0, 03130Z1, 03130Z6, 03130Z7, 03130Z8, 03130Z9, 03130ZB, 03130ZC, 03130ZD, 03130ZF, 03130ZJ, 03130ZK, 03130ZM, 03130ZW, 0314090, 0314091, 0314094, 0314096, 0314097, 0314098, 0314099, 031409B, 031409C, 031409D, 031409J, 031409K, 03140A1, 03140A4, 03140A6, 03140A7, 03140A8, 03140A9, 03140AB, 03140AC, 03140AF, 03140AK, 03140AW, 03140J6, 03140J7, 03140J8, 03140J9, 03140JB, 03140JC, 03140K1, 03140K6, 03140K7, 03140K8, 03140K9, 03140KB, 03140KC, 03140KD, 03140KF, 03140KJ, 03140KK, 03140Z0, 03140Z1, 03140Z4, 03140Z6, 03140Z7, 03140Z8, 03140Z9, 03140ZB, 03140ZC, 03140ZD, 03140ZF, 03140ZJ, 03140ZK, 03140ZM, 03140ZN, 03140ZW, 0315096, 0315097, 0315098, 0315099, 031509B, 031509C, 03150A6, 03150A7, 03150A8, 03150A9, 03150AB, 03150AC, 03150J6, 03150J7, 03150J8, 03150J9, 03150JB, 03150JC, 03150K6, 03150K7, 03150K8, 03150K9, 03150KB, 03150KC, 03150Z6, 03150Z7, 03150Z8, 03150Z9, 03150ZB, 03150ZC, 0316090, 0316091, 0316094, 0316096, 0316097, 0316098, 0316099, 031609B, 031609C, 031609D, 031609F, 031609K, 031609W, 03160A0, 03160A1, 03160A2, 03160A4, 03160A6, 03160A7, 03160A8, 03160A9, 03160AB, 03160AC, 03160AD, 03160AF, 03160J6, 03160J7, 03160J8, 03160J9, 03160JB, 03160JC, 03160K6, 03160K7, 03160K8, 03160K9, 03160KB, 03160KC, 03160Z6, 03160Z7, 03160Z8, 03160Z9, 03160ZB, 03160ZC, 03C00ZZ, 03C04ZZ, 03C10ZZ, 03C14ZZ, 03C20ZZ, 03C24ZZ, 03C30ZZ, 03C34ZZ, 03C40ZZ, 03C44ZZ

Clinical condition	ICD code for clinical condition
**PVD (continued)**	0410096, 0410097, 0410098, 0410099, 041009B, 041009C, 041009D, 041009F, 041009G, 041009H, 041009J, 041009K, 041009Q, 041009R, 04100A6, 04100A7, 04100A8, 04100A9, 04100AB, 04100AC, 04100AD, 04100AF, 04100AG, 04100AH, 04100AJ, 04100AK, 04100AQ, 04100AR, 04100J6, 04100J7, 04100J8, 04100J9, 04100JB, 04100JC, 04100JD, 04100JF, 04100JG, 04100JH, 04100JJ, 04100JK, 04100JQ, 04100JR, 04100K6, 04100K7, 04100K8, 04100K9, 04100KB, 04100KC, 04100KD, 04100KF, 04100KG, 04100KH, 04100KJ, 04100KK, 04100KQ, 04100KR, 04100Z6, 04100Z7, 04100Z8, 04100Z9, 04100ZB, 04100ZC, 04100ZD, 04100ZF, 04100ZG, 04100ZH, 04100ZJ, 04100ZK, 04100ZQ, 04100ZR, 0410491, 0410493, 0410496, 0410497, 0410498, 0410499, 041049B, 041049C, 041049D, 041049F, 041049G, 041049H, 041049J, 041049K, 041049Q, 041049R, 04104A2, 04104A6, 04104A7, 04104A8, 04104A9, 04104AB, 04104AC, 04104AD, 04104AF, 04104AG, 04104AH, 04104AJ, 04104AK, 04104AQ, 04104AR, 04104J0, 04104J1, 04104J2, 04104J4, 04104J5, 04104J6, 04104J7, 04104J8, 04104J9, 04104JB, 04104JC, 04104JD, 04104JF, 04104JG, 04104JH, 04104JJ, 04104JK, 04104JQ, 04104JR, 04104K6, 04104K7, 04104K8, 04104K9, 04104KB, 04104KC, 04104KD, 04104KF, 04104KG, 04104KH, 04104KJ, 04104KK, 04104KQ, 04104KR, 04104Z2, 04104Z4, 04104Z6, 04104Z7, 04104Z8, 04104Z9, 04104ZB, 04104ZC, 04104ZD, 04104ZF, 04104ZG, 04104ZH, 04104ZJ, 04104ZK, 04104ZQ, 04104ZR, 041C091, 041C092, 041C093, 041C096, 041C097, 041C098, 041C099, 041C09B, 041C09C, 041C09D, 041C09F, 041C09G, 041C09H, 041C09J, 041C09K, 041C09Q, 041C09R, 041C0A0, 041C0A2, 041C0A3, 041C0A5, 041C0AH, 041C0AJ, 041C0AK, 041C0AQ, 041C0AR, 041C0J0, 041C0J1, 041C0J2, 041C0J3, 041C0J4, 041C0J5, 041C0J6, 041C0J7, 041C0J8, 041C0J9, 041C0JB, 041C0JC, 041C0JD, 041C0JF, 041C0JG, 041C0JH, 041C0JJ, 041C0JK, 041C0JQ, 041C0JR, 041C0K0, 041C0K1, 041C0K2, 041C0K3, 041C0K6, 041C0K7, 041C0K8, 041C0K9, 041C0KB, 041C0KC, 041C0KD, 041C0KF, 041C0KG, 041C0KH, 041C0KJ, 041C0KK, 041C0KQ, 041C0KR, 041C0Z0, 041C0Z1, 041C0Z2, 041C0Z3, 041C0Z4, 041C0Z5, 041C0Z6, 041C0Z7, 041C0Z8, 041C0Z9, 041C0ZB, 041C0ZC, 041C0ZD, 041C0ZF, 041C0ZG, 041C0ZH, 041C0ZJ, 041C0ZK, 041C0ZQ, 041C0ZR, 041C496, 041C497, 041C498, 041C499, 041C49B, 041C49C, 041C49D, 041C49F, 041C49G, 041C49H, 041C49J, 041C49K, 041C49Q, 041C4A6, 041C4A7, 041C4A8, 041C4A9, 041C4AB, 041C4AC, 041C4AD, 041C4AF, 041C4AG, 041C4AH, 041C4AJ, 041C4AK, 041C4AQ, 041C4J0, 041C4J2, 041C4J4, 041C4J5, 041C4J6, 041C4J7, 041C4J8, 041C4J9, 041C4JB, 041C4JC, 041C4JD, 041C4JF, 041C4JG, 041C4JH, 041C4JJ, 041C4JK, 041C4JQ, 041C4K6, 041C4K7, 041C4K8, 041C4K9, 041C4KB, 041C4KC, 041C4KD, 041C4KF, 041C4KG, 041C4KH, 041C4KJ, 041C4KK, 041C4KQ, 041C4Z6, 041C4Z7, 041C4Z8, 041C4Z9, 041C4ZB, 041C4ZC, 041C4ZD, 041C4ZF, 041C4ZG, 041C4ZH, 041C4ZJ, 041C4ZK, 041C4ZQ, 041C4ZR

Clinical condition	ICD code for clinical condition
**PVD (continued)**	041D092, 041D094, 041D097, 041D098, 041D099, 041D09B, 041D09C, 041D09D, 041D09F, 041D09G, 041D09H, 041D09J, 041D09K, 041D09Q, 041D09R, 041D0A0, 041D0A1, 041D0A2, 041D0A4, 041D0A5, 041D0A6, 041D0A7, 041D0A8, 041D0A9, 041D0AB, 041D0AC, 041D0AD, 041D0AF, 041D0AG, 041D0AH, 041D0AJ, 041D0AK, 041D0AQ, 041D0J0, 041D0J1, 041D0J2, 041D0J3, 041D0J4, 041D0J5, 041D0J6, 041D0J7, 041D0J8, 041D0J9, 041D0JB, 041D0JC, 041D0JD, 041D0JF, 041D0JG, 041D0JH, 041D0JJ, 041D0JK, 041D0JQ, 041D0JR, 041D0K0, 041D0K1, 041D0K2, 041D0K4, 041D0K5, 041D0K6, 041D0K7, 041D0K8, 041D0K9, 041D0KB, 041D0KC, 041D0KD, 041D0KF, 041D0KG, 041D0KH, 041D0KJ, 041D0KK, 041D0KQ, 041D0Z0, 041D0Z1, 041D0Z2, 041D0Z3, 041D0Z4, 041D0Z5, 041D0Z6, 041D0Z7, 041D0Z8, 041D0Z9, 041D0ZB, 041D0ZC, 041D0ZD, 041D0ZF, 041D0ZG, 041D0ZH, 041D0ZJ, 041D0ZK, 041D0ZQ, 041D0ZR, 041D496, 041D497, 041D498, 041D499, 041D49B, 041D49C, 041D49D, 041D49F, 041D49G, 041D49H, 041D49J, 041D49K, 041D49Q, 041D4A6, 041D4A7, 041D4A8, 041D4A9, 041D4AB, 041D4AC, 041D4AD, 041D4AF, 041D4AG, 041D4AH, 041D4AJ, 041D4AK, 041D4AQ, 041D4J0, 041D4J1, 041D4J6, 041D4J7, 041D4J8, 041D4J9, 041D4JB, 041D4JC, 041D4JD, 041D4JF, 041D4JG, 041D4JH, 041D4JJ, 041D4JK, 041D4JQ, 041D4JR, 041D4K7, 041D4K8, 041D4K9, 041D4KB, 041D4KC, 041D4KD, 041D4KF, 041D4KG, 041D4KH, 041D4KJ, 041D4KK, 041D4KQ, 041D4Z3, 041D4Z6, 041D4Z7, 041D4Z8, 041D4Z9, 041D4ZB, 041D4ZC, 041D4ZD, 041D4ZF, 041D4ZG, 041D4ZH, 041D4ZJ, 041D4ZK, 041D4ZQ, 041D4ZR, 041E099, 041E09B, 041E09C, 041E09D, 041E09F, 041E09G, 041E09H, 041E09J, 041E09K, 041E09P, 041E09Q, 041E0A9, 041E0AB, 041E0AC, 041E0AD, 041E0AF, 041E0AG, 041E0AH, 041E0AJ, 041E0AK, 041E0AP, 041E0AQ, 041E0J9, 041E0JB, 041E0JC, 041E0JD, 041E0JF, 041E0JG, 041E0JH, 041E0JJ, 041E0JK, 041E0JP, 041E0JQ, 041E0K9, 041E0KB, 041E0KC, 041E0KD, 041E0KF, 041E0KG, 041E0KH, 041E0KJ, 041E0KK, 041E0KP, 041E0KQ, 041E0Z9, 041E0ZB, 041E0ZC, 041E0ZD, 041E0ZF, 041E0ZG, 041E0ZH, 041E0ZJ, 041E0ZK, 041E0ZP, 041E0ZQ, 041E499, 041E49B, 041E49C, 041E49D, 041E49F, 041E49G, 041E49H, 041E49J, 041E49K, 041E49P, 041E49Q, 041E4A9, 041E4AB, 041E4AC, 041E4AD, 041E4AF, 041E4AG, 041E4AH, 041E4AJ, 041E4AK, 041E4AP, 041E4AQ, 041E4J9, 041E4JB, 041E4JC, 041E4JD, 041E4JF, 041E4JG, 041E4JH, 041E4JJ, 041E4JK, 041E4JP, 041E4JQ, 041E4K9, 041E4KB, 041E4KC, 041E4KD, 041E4KF, 041E4KG, 041E4KH, 041E4KJ, 041E4KK, 041E4KP, 041E4KQ, 041E4Z9, 041E4ZB, 041E4ZC, 041E4ZD, 041E4ZF, 041E4ZG, 041E4ZH, 041E4ZJ, 041E4ZK, 041E4ZP, 041E4ZQ

Clinical condition	ICD code for clinical condition
**PVD (continued)**	041F099, 041F09B, 041F09C, 041F09D, 041F09F, 041F09G, 041F09H, 041F09J, 041F09K, 041F09P, 041F09Q, 041F0A9, 041F0AB, 041F0AC, 041F0AD, 041F0AF, 041F0AG, 041F0AH, 041F0AJ, 041F0AK, 041F0AP, 041F0AQ, 041F0J9, 041F0JB, 041F0JC, 041F0JD, 041F0JF, 041F0JG, 041F0JH, 041F0JJ, 041F0JK, 041F0JP, 041F0JQ, 041F0K9, 041F0KB, 041F0KC, 041F0KD, 041F0KF, 041F0KG, 041F0KH, 041F0KJ, 041F0KK, 041F0KP, 041F0KQ, 041F0Z9, 041F0ZB, 041F0ZC, 041F0ZD, 041F0ZF, 041F0ZG, 041F0ZH, 041F0ZJ, 041F0ZK, 041F0ZP, 041F0ZQ, 041F499, 041F49B, 041F49C, 041F49D, 041F49F, 041F49G, 041F49H, 041F49J, 041F49K, 041F49P, 041F49Q, 041F4A9, 041F4AB, 041F4AC, 041F4AD, 041F4AF, 041F4AG, 041F4AH, 041F4AJ, 041F4AK, 041F4AP, 041F4AQ, 041F4J9, 041F4JB, 041F4JC, 041F4JD, 041F4JF, 041F4JG, 041F4JH, 041F4JJ, 041F4JK, 041F4JP, 041F4JQ, 041F4K9, 041F4KB, 041F4KC, 041F4KD, 041F4KF, 041F4KG, 041F4KH, 041F4KJ, 041F4KK, 041F4KP, 041F4KQ, 041F4Z9, 041F4ZB, 041F4ZC, 041F4ZD, 041F4ZF, 041F4ZG, 041F4ZH, 041F4ZJ, 041F4ZK, 041F4ZP, 041F4ZQ, 041H099, 041H09B, 041H09C, 041H09D, 041H09F, 041H09G, 041H09H, 041H09J, 041H09K, 041H09P, 041H09Q, 041H0A9, 041H0AB, 041H0AC, 041H0AD, 041H0AF, 041H0AG, 041H0AH, 041H0AJ, 041H0AK, 041H0AP, 041H0AQ, 041H0J9, 041H0JB, 041H0JC, 041H0JD, 041H0JF, 041H0JG, 041H0JH, 041H0JJ, 041H0JK, 041H0JP, 041H0JQ, 041H0K9, 041H0KB, 041H0KC, 041H0KD, 041H0KF, 041H0KG, 041H0KH, 041H0KJ, 041H0KK, 041H0KP, 041H0KQ, 041H0Z9, 041H0ZB, 041H0ZC, 041H0ZD, 041H0ZF, 041H0ZG, 041H0ZH, 041H0ZJ, 041H0ZK, 041H0ZP, 041H0ZQ, 041H499, 041H49B, 041H49C, 041H49D, 041H49F, 041H49G, 041H49H, 041H49J, 041H49K, 041H49P, 041H49Q, 041H4A9, 041H4AB, 041H4AC, 041H4AD, 041H4AF, 041H4AG, 041H4AH, 041H4AJ, 041H4AK, 041H4AP, 041H4AQ, 041H4J9, 041H4JB, 041H4JC, 041H4JD, 041H4JF, 041H4JG, 041H4JH, 041H4JJ, 041H4JK, 041H4JP, 041H4JQ, 041H4K9, 041H4KB, 041H4KC, 041H4KD, 041H4KF, 041H4KG, 041H4KH, 041H4KJ, 041H4KK, 041H4KP, 041H4KQ, 041H4Z9, 041H4ZB, 041H4ZC, 041H4ZD, 041H4ZF, 041H4ZG, 041H4ZH, 041H4ZJ, 041H4ZK, 041H4ZP, 041H4ZQ

Clinical condition	ICD code for clinical condition
**PVD (continued)**	041J099, 041J09B, 041J09C, 041J09D, 041J09F, 041J09G, 041J09H, 041J09J, 041J09K, 041J09P, 041J09Q, 041J0A9, 041J0AB, 041J0AC, 041J0AD, 041J0AF, 041J0AG, 041J0AH, 041J0AJ, 041J0AK, 041J0AP, 041J0AQ, 041J0J9, 041J0JB, 041J0JC, 041J0JD, 041J0JF, 041J0JG, 041J0JH, 041J0JJ, 041J0JK, 041J0JP, 041J0JQ, 041J0K9, 041J0KB, 041J0KC, 041J0KD, 041J0KF, 041J0KG, 041J0KH, 041J0KJ, 041J0KK, 041J0KP, 041J0KQ, 041J0Z9, 041J0ZB, 041J0ZC, 041J0ZD, 041J0ZF, 041J0ZG, 041J0ZH, 041J0ZJ, 041J0ZK, 041J0ZP, 041J0ZQ, 041J499, 041J49B, 041J49C, 041J49D, 041J49F, 041J49G, 041J49H, 041J49J, 041J49K, 041J49P, 041J49Q, 041J4A9, 041J4AB, 041J4AC, 041J4AD, 041J4AF, 041J4AG, 041J4AH, 041J4AJ, 041J4AK, 041J4AP, 041J4AQ, 041J4J9, 041J4JB, 041J4JC, 041J4JD, 041J4JF, 041J4JG, 041J4JH, 041J4JJ, 041J4JK, 041J4JP, 041J4JQ, 041J4K9, 041J4KB, 041J4KC, 041J4KD, 041J4KF, 041J4KG, 041J4KH, 041J4KJ, 041J4KK, 041J4KP, 041J4KQ, 041J4Z9, 041J4ZB, 041J4ZC, 041J4ZD, 041J4ZF, 041J4ZG, 041J4ZH, 041J4ZJ, 041J4ZK, 041J4ZP, 041J4ZQ, 041K09H, 041K09J, 041K09K, 041K09L, 041K09M, 041K09N, 041K09P, 041K09Q, 041K0AH, 041K0AJ, 041K0AK, 041K0AL, 041K0AM, 041K0AN, 041K0AP, 041K0AQ, 041K0JH, 041K0JJ, 041K0JK, 041K0JL, 041K0JM, 041K0JN, 041K0JP, 041K0JQ, 041K0KH, 041K0KJ, 041K0KK, 041K0KL, 041K0KM, 041K0KN, 041K0KP, 041K0KQ, 041K0ZH, 041K0ZJ, 041K0ZK, 041K0ZL, 041K0ZM, 041K0ZN, 041K0ZP, 041K0ZQ, 041K49H, 041K49J, 041K49K, 041K49L, 041K49M, 041K49N, 041K49P, 041K49Q, 041K4AH, 041K4AJ, 041K4AK, 041K4AL, 041K4AM, 041K4AN, 041K4AP, 041K4AQ, 041K4JH, 041K4JJ, 041K4JK, 041K4JL, 041K4JM, 041K4JN, 041K4JP, 041K4JQ, 041K4KH, 041K4KJ, 041K4KK, 041K4KL, 041K4KM, 041K4KN, 041K4KP, 041K4KQ, 041K4KS, 041K4ZH, 041K4ZJ, 041K4ZK, 041K4ZL, 041K4ZM, 041K4ZN, 041K4ZP, 041K4ZQ, 041K4ZS, 041L09H, 041L09J, 041L09K, 041L09L, 041L09M, 041L09N, 041L09P, 041L09Q, 041L0AH, 041L0AJ, 041L0AK, 041L0AL, 041L0AM, 041L0AN, 041L0AP, 041L0AQ, 041L0JH, 041L0JJ, 041L0JK, 041L0JL, 041L0JM, 041L0JN, 041L0JP, 041L0JQ, 041L0KH, 041L0KJ, 041L0KK, 041L0KL, 041L0KM, 041L0KN, 041L0KP, 041L0KQ, 041L0ZH, 041L0ZJ, 041L0ZK, 041L0ZL, 041L0ZM, 041L0ZN, 041L0ZP, 041L0ZQ, 041L49H, 041L49J, 041L49K, 041L49L, 041L49M, 041L49N, 041L49P, 041L49Q, 041L49S, 041L4AH, 041L4AJ, 041L4AK, 041L4AL, 041L4AM, 041L4AN, 041L4AP, 041L4AQ, 041L4AS, 041L4JH, 041L4JJ, 041L4JK, 041L4JL, 041L4JM, 041L4JN, 041L4JP, 041L4JQ, 041L4JS, 041L4KH, 041L4KJ, 041L4KK, 041L4KL, 041L4KM, 041L4KN, 041L4KP, 041L4KQ, 041L4ZH, 041L4ZJ, 041L4ZK, 041L4ZL, 041L4ZM, 041L4ZN, 041L4ZP, 041L4ZQ, 041M09L, 041M09M, 041M09P, 041M09Q, 041M0AL, 041M0AM, 041M0AP, 041M0AQ, 041M0JL, 041M0JM, 041M0JP, 041M0JQ, 041M0KL, 041M0KM, 041M0KP, 041M0KQ, 041M0ZL, 041M0ZM, 041M0ZP, 041M0ZQ, 041M49L, 041M49M, 041M49P, 041M49Q, 041M4AL, 041M4AM, 041M4AP, 041M4AQ, 041M4JL, 041M4JM, 041M4JP, 041M4JQ, 041M4JS, 041M4KL, 041M4KM, 041M4KP, 041M4KQ, 041M4ZL, 041M4ZM, 041M4ZP, 041M4ZQ

Clinical condition	ICD code for clinical condition
**PVD (continued)**	041N09L, 041N09M, 041N09P, 041N09Q, 041N0AL, 041N0AM, 041N0AP, 041N0AQ, 041N0JL, 041N0JM, 041N0JP, 041N0JQ, 041N0KL, 041N0KM, 041N0KP, 041N0KQ, 041N0ZL, 041N0ZM, 041N0ZP, 041N0ZQ, 041N49L, 041N49M, 041N49P, 041N49Q, 041N4AL, 041N4AM, 041N4AP, 041N4AQ, 041N4JL, 041N4JM, 041N4JP, 041N4JQ, 041N4JS, 041N4KL, 041N4KM, 041N4KP, 041N4KQ, 041N4KS, 041N4ZL, 041N4ZM, 041N4ZP, 041N4ZQ, 041T09P, 041T09Q, 041T0AP, 041T0AQ, 041T0JP, 041T0JQ, 041T0KP, 041T0KQ, 041T0KS, 041T0ZP, 041T0ZQ, 041T0ZS, 041T49P, 041T49Q, 041T4AP, 041T4AQ, 041T4JP, 041T4JQ, 041T4KP, 041T4KQ, 041T4ZP, 041T4ZQ, 041T4ZS, 041U09P, 041U09Q, 041U0AP, 041U0AQ, 041U0JP, 041U0JQ, 041U0JS, 041U0KP, 041U0KQ, 041U0ZP, 041U0ZQ, 041U49P, 041U49Q, 041U4AP, 041U4AQ, 041U4JP, 041U4JQ, 041U4KP, 041U4KQ, 041U4ZP, 041U4ZQ, 041V09P, 041V09Q, 041V0AP, 041V0AQ, 041V0AS, 041V0JP, 041V0JQ, 041V0KP, 041V0KQ, 041V0ZP, 041V0ZQ, 041V49P, 041V49Q, 041V4AP, 041V4AQ, 041V4JP, 041V4JQ, 041V4JS, 041V4KP, 041V4KQ, 041V4ZP, 041V4ZQ, 041W09P, 041W09Q, 041W0AP, 041W0AQ, 041W0JP, 041W0JQ, 041W0JS, 041W0KP, 041W0KQ, 041W0ZP, 041W0ZQ, 041W49P, 041W49Q, 041W49S, 041W4AP, 041W4AQ, 041W4JP, 041W4JQ, 041W4KP, 041W4KQ, 041W4ZP, 041W4ZQ, 047C041, 047C04Z, 047C0D1, 047C0DZ, 047C0Z1, 047C0ZZ, 047C341, 047C34Z, 047C3D1, 047C3DZ, 047C3Z1, 047C3ZZ, 047C441, 047C44Z, 047C4D1, 047C4DZ, 047C4Z1, 047C4ZZ, 047D041, 047D04Z, 047D0D1, 047D0DZ, 047D0Z1, 047D0ZZ, 047D341, 047D34Z, 047D3D1, 047D3DZ, 047D3Z1, 047D3ZZ, 047D441, 047D44Z, 047D4D1, 047D4DZ, 047D4Z1, 047D4ZZ, 047E04Z, 047E0D1, 047E0D6, 047E0DZ, 047E0Z1, 047E0ZZ, 047E341, 047E34Z, 047E3D1, 047E3DZ, 047E3Z1, 047E3ZZ, 047E441, 047E446, 047E44Z, 047E4D1, 047E4D6, 047E4DZ, 047E4Z1, 047E4ZZ, 047F041, 047F04Z, 047F0D1, 047F0DZ, 047F0Z1, 047F0ZZ, 047F341, 047F34Z, 047F3D1, 047F3DZ, 047F3Z1, 047F3ZZ, 047F441, 047F44Z, 047F4D1, 047F4DZ, 047F4Z1, 047F4ZZ, 047H041, 047H04Z, 047H0D1, 047H0DZ, 047H0Z1, 047H0ZZ, 047H341, 047H34Z, 047H3D1, 047H3DZ, 047H3Z1, 047H3ZZ, 047H441, 047H446, 047H44Z, 047H4D1, 047H4D6, 047H4DZ, 047H4Z1, 047H4ZZ, 047J041, 047J04Z, 047J0D1, 047J0DZ, 047J0Z1, 047J0ZZ, 047J341, 047J34Z, 047J3D1, 047J3DZ, 047J3Z1, 047J3ZZ, 047J441, 047J44Z, 047J4D1, 047J4DZ, 047J4Z1, 047J4ZZ, 047K041, 047K04Z, 047K0D1, 047K0DZ, 047K0Z1, 047K0ZZ, 047K341, 047K34Z, 047K3D1, 047K3DZ, 047K3Z1, 047K3ZZ, 047K441, 047K44Z, 047K4D1, 047K4DZ, 047K4Z1, 047K4ZZ, 047L041, 047L04Z, 047L0D1, 047L0DZ, 047L0Z1, 047L0ZZ, 047L341, 047L34Z, 047L3D1, 047L3DZ, 047L3Z1, 047L3ZZ, 047L441, 047L44Z, 047L4D1, 047L4DZ, 047L4Z1, 047L4ZZ, 047M041, 047M04Z, 047M0D1, 047M0DZ, 047M0Z1, 047M0ZZ, 047M341, 047M34Z, 047M3D1, 047M3DZ, 047M3Z1, 047M3ZZ, 047M441, 047M44Z, 047M4D1, 047M4DZ, 047M4Z1, 047M4ZZ, 047N041, 047N04Z, 047N0D1, 047N0DZ, 047N0Z1, 047N0ZZ, 047N341, 047N34Z, 047N3D1, 047N3DZ, 047N3Z1, 047N3ZZ, 047N441, 047N44Z, 047N4D1, 047N4DZ, 047N4Z1, 047N4ZZ, 047P041, 047P04Z, 047P0D1, 047P0DZ, 047P0Z1, 047P0ZZ, 047P341, 047P34Z, 047P3D1, 047P3DZ, 047P3Z1, 047P3ZZ, 047P441, 047P44Z, 047P4D1, 047P4DZ, 047P4Z1, 047P4ZZ

Clinical condition	ICD code for clinical condition
**PVD (continued)**	047Q041, 047Q04Z, 047Q0D1, 047Q0DZ, 047Q0Z1, 047Q0ZZ, 047Q341, 047Q34Z, 047Q3D1, 047Q3DZ, 047Q3Z1, 047Q3ZZ, 047Q441, 047Q44Z, 047Q4D1, 047Q4DZ, 047Q4Z1, 047Q4ZZ, 047R041, 047R04Z, 047R0D1, 047R0DZ, 047R0Z1, 047R0ZZ, 047R341, 047R34Z, 047R3D1, 047R3DZ, 047R3Z1, 047R3ZZ, 047R441, 047R44Z, 047R4D1, 047R4DZ, 047R4Z1, 047R4ZZ, 047S041, 047S04Z, 047S0D1, 047S0DZ, 047S0Z1, 047S0ZZ, 047S341, 047S34Z, 047S3D1, 047S3DZ, 047S3Z1, 047S3ZZ, 047S441, 047S44Z, 047S4D1, 047S4DZ, 047S4Z1, 047S4ZZ, 047T041, 047T04Z, 047T0D1, 047T0DZ, 047T0Z1, 047T0ZZ, 047T341, 047T34Z, 047T3D1, 047T3DZ, 047T3Z1, 047T3ZZ, 047T441, 047T44Z, 047T4D1, 047T4DZ, 047T4Z1, 047T4ZZ, 047U041, 047U04Z, 047U0D1, 047U0DZ, 047U0Z1, 047U0ZZ, 047U341, 047U34Z, 047U3D1, 047U3DZ, 047U3Z1, 047U3ZZ, 047U441, 047U44Z, 047U4D1, 047U4DZ, 047U4Z1, 047U4ZZ, 047V041, 047V04Z, 047V0D1, 047V0DZ, 047V0Z1, 047V0ZZ, 047V341, 047V34Z, 047V3D1, 047V3DZ, 047V3Z1, 047V3ZZ, 047V441, 047V44Z, 047V4D1, 047V4DZ, 047V4Z1, 047V4ZZ, 047W041, 047W04Z, 047W0D1, 047W0DZ, 047W0Z1, 047W0ZZ, 047W341, 047W34Z, 047W3D1, 047W3DZ, 047W3Z1, 047W3ZZ, 047W441, 047W44Z, 047W4D1, 047W4DZ, 047W4Z1, 047W4ZZ, 047Y04Z, 047Y0DZ, 047Y0ZZ, 047Y34Z, 047Y3DZ, 047Y3ZZ, 047Y44Z, 047Y4DZ, 047Y4ZZ, 04C00ZZ, 04C04ZZ, 04C10ZZ, 04C14ZZ, 04C50ZZ, 04C54ZZ, 04C90ZZ, 04C94ZZ, 04CA0ZZ, 04CA4ZZ, 04CC0ZZ, 04CC4ZZ, 04CD0ZZ, 04CD4ZZ, 04CE0ZZ, 04CE4ZZ, 04CF0ZZ, 04CF4ZZ, 04CH0ZZ, 04CH4ZZ, 04CJ0ZZ, 04CJ4ZZ, 04CK0ZZ, 04CK3ZZ, 04CK4ZZ, 04CL0ZZ, 04CL3ZZ, 04CL4ZZ, 04CM0ZZ, 04CM3ZZ, 04CM4ZZ, 04CN0ZZ, 04CN3ZZ, 04CN4ZZ, 04CP0ZZ, 04CP3ZZ, 04CP4ZZ, 04CQ0ZZ, 04CQ3ZZ, 04CQ4ZZ, 04CR0ZZ, 04CR3ZZ, 04CR4ZZ, 04CS0ZZ, 04CS3ZZ, 04CS4ZZ, 04CT0ZZ, 04CT3ZZ, 04CT4ZZ, 04CU0ZZ, 04CU3ZZ, 04CU4ZZ, 04RK07Z, 04RK0JZ, 04RK0KZ, 04RK47Z, 04RK4JZ, 04RK4KZ, 04RL07Z, 04RL0JZ, 04RL0KZ, 04RL47Z, 04RL4JZ, 04RL4KZ, 04RM07Z, 04RM0JZ, 04RM0KZ, 04RM47Z, 04RM4JZ, 04RM4KZ, 04RN07Z, 04RN0JZ, 04RN0KZ, 04RN47Z, 04RN4JZ, 04RN4KZ, 04RP07Z, 04RP0JZ, 04RP0KZ, 04RP47Z, 04RP4JZ, 04RP4KZ, 04RQ07Z, 04RQ0JZ, 04RQ0KZ, 04RQ47Z, 04RQ4JZ, 04RQ4KZ, 04RR07Z, 04RR0JZ, 04RR0KZ, 04RR47Z, 04RR4JZ, 04RR4KZ, 04RS07Z, 04RS0JZ, 04RS0KZ, 04RS47Z, 04RS4JZ, 04RS4KZ, 04RT07Z, 04RT0JZ, 04RT0KZ, 04RT47Z, 04RT4JZ, 04RT4KZ, 04RU07Z, 04RU0JZ, 04RU0KZ, 04RU47Z, 04RU4JZ, 04RU4KZ, 04RV07Z, 04RV0JZ, 04RV0KZ, 04RV47Z, 04RV4JZ, 04RV4KZ, 04RW07Z, 04RW0JZ, 04RW0KZ, 04RW47Z, 04RW4JZ, 04RW4KZ, 04RY07Z, 04RY0JZ, 04RY0KZ, 04RY47Z, 04RY4JZ, 04RY4KZ, 34812, 34813, 34820, 34900, 37220, 37221, 37222, 37223, 37224, 37225, 37226, 37227, 37228, 37229, 37230, 37231, 37232, 37233, 37234, 37235, 37238, 37239, 38.16, 38.18, 38.38, 38.48, 38.68, 39.25, 39.26, 39.29, 39.50, 39.90, 440.1, 440.2, 440.20, 440.21, 440.22, 440.23, 440.24, 440.29, 440.3, 440.30, 440.31, 440.32, 440.4, 443, 443.8, 443.81, 443.89, 443.9, 444, 444.2, 444.21, 445.0, 445.00, 445.01, 445.02, 445.03, 445.04, 445.05, 445.06, 445.07, 445.08, 445.09, 445.8, 445.81, 445.89, Z95.820, Z95.828, Z98.62

Clinical condition	ICD code for clinical condition
**PVD (concluded)**	35102, 35103, 35131, 35132, 35141, 35142, 35151, 35152, 35302, 35303, 35304, 35305, 35306, 35311, 35321, 35331, 35341, 35351, 35355, 35361, 35363, 35371, 35372, 35381, 35454, 35456, 35459, 35470, 35473, 35474, 35480, 35482, 35483, 35485, 35490, 35491, 35492, 35493, 35495, 35511, 35512, 35515, 35516, 35518, 35521, 35522, 35523, 35525, 35526, 35531, 35533, 35535, 35536, 35537, 35538, 35539, 35540, 35541, 35546, 35548, 35549, 35551, 35556, 35558, 35560, 35563, 35565, 35566, 35570, 35571, 35582, 35583, 35585, 35587, 35612, 35616, 35621, 35623, 35626, 35631, 35632, 35633, 35634, 35636, 35637, 35638, 35641, 35645, 35646, 35647, 35650, 35651, 35654, 35656, 35661, 35663, 35665, 35666, 35671, 35875, 35876, 35879, 35881, 35883, 35884, I70.1, I70.2, I70.20, I70.201, I70.202, I70.203, I70.208, I70.209, I70.21, I70.211, I70.212, I70.213, I70.218, I70.219, I70.22, I70.221, I70.222, I70.223, I70.228, I70.229, I70.23, I70.231, I70.232, I70.233, I70.234, I70.235, I70.238, I70.239, I70.24, I70.241, I70.242, I70.243, I70.244, I70.245, I70.248, I70.249, I70.25, I70.26, I70.261, I70.262, I70.263, I70.268, I70.269, I70.29, I70.291, I70.292, I70.293, I70.298, I70.299, I70.3, I70.30, I70.301, I70.302, I70.303, I70.308, I70.309, I70.31, I70.311, I70.312, I70.313, I70.318, I70.319, I70.32, I70.321, I70.322, I70.323, I70.328, I70.329, I70.33, I70.331, I70.332, I70.333, I70.334, I70.335, I70.338, I70.339, I70.34, I70.341, I70.342, I70.343, I70.344, I70.345, I70.348, I70.349, I70.35, I70.36, I70.361, I70.362, I70.363, I70.368, I70.369, I70.39, I70.391, I70.392, I70.393, I70.398, I70.399, I70.4, I70.40, I70.401, I70.402, I70.403, I70.408, I70.409, I70.41, I70.411, I70.412, I70.413, I70.418, I70.419, I70.42, I70.421, I70.422, I70.423, I70.428, I70.429, I70.43, I70.431, I70.432, I70.433, I70.434, I70.435, I70.438, I70.439, I70.44, I70.441, I70.442, I70.443, I70.444, I70.445, I70.448, I70.449, I70.45, I70.46, I70.461, I70.462, I70.463, I70.468, I70.469, I70.49, I70.491, I70.492, I70.493, I70.498, I70.499, I70.5, I70.50, I70.501, I70.502, I70.503, I70.508, I70.509, I70.51, I70.511, I70.512, I70.513, I70.518, I70.519, I70.52, I70.521, I70.522, I70.523, I70.528, I70.529, I70.53, I70.531, I70.532, I70.533, I70.534, I70.535, I70.538, I70.539, I70.54, I70.541, I70.542, I70.543, I70.544, I70.545, I70.548, I70.549, I70.55, I70.56, I70.561, I70.562, I70.563, I70.568, I70.569, I70.59, I70.591, I70.592, I70.593, I70.598, I70.599, I70.6, I70.60, I70.601, I70.602, I70.603, I70.608, I70.609, I70.61, I70.611, I70.612, I70.613, I70.618, I70.619, I70.62, I70.621, I70.622, I70.623, I70.628, I70.629, I70.63, I70.631, I70.632, I70.633, I70.634, I70.635, I70.638, I70.639, I70.64, I70.641, I70.642, I70.643, I70.644, I70.645, I70.648, I70.649, I70.65, I70.66, I70.661, I70.662, I70.663, I70.668, I70.669, I70.69, I70.691, I70.692, I70.693, I70.698, I70.699, I70.7, I70.70, I70.701, I70.702, I70.703, I70.708, I70.709, I70.71, I70.711, I70.712, I70.713, I70.718, I70.719, I70.72, I70.721, I70.722, I70.723, I70.728, I70.729, I70.73, I70.731, I70.732, I70.733, I70.734, I70.735, I70.738, I70.739, I70.74, I70.741, I70.742, I70.743, I70.744, I70.745, I70.748, I70.749, I70.75, I70.76, I70.761, I70.762, I70.763, I70.768, I70.769, I70.79, I70.791, I70.792, I70.793, I70.798, I70.799, I70.92, I73.89, I73.9, I75.011, I75.012, I75.013, I75.019, I75.021, I75.022, I75.023, I75.029, I75.81, I75.89

Table S1b. Recurrent ASCVD events: Clinical conditions with associated ICD codes

ASCVD = atherosclerotic cardiovascular disease; CABG = coronary artery bypass graft; MI = myocardial infarction; PCI = percutaneous coronary intervention; PVD = peripheral vascular disease; TIA = transient ischemic attack.

Clinical condition	ICD code for clinical condition
**Acute Ischemic Heart Disease**	3604, 3609, 3E07017, 3E070PZ, 411.81, 92995, 92996, C9603, I24.0, I24.9, X2C3361
**Ischemic Stroke**	433.01, 433.11, 433.21, 433.31, 433.41, 433.51, 433.61, 433.71, 433.81, 433.91, 434.01, 434.11, 434.91, I63, I63.0, I63.00, I63.01, I63.011, I63.012, I63.013, I63.019, I63.02, I63.03, I63.031, I63.032, I63.033, I63.039, I63.09, I63.1, I63.10, I63.111, I63.112, I63.113, I63.119, I63.12, I63.131, I63.132, I63.133, I63.139, I63.19, I63.2, I63.20, I63.21, I63.211, I63.212, I63.213, I63.219, I63.22, I63.23, I63.231, I63.232, I63.233, I63.239, I63.29, I63.3, I63.30, I63.31, I63.311, I63.312, I63.313, I63.319, I63.32, I63.321, I63.322, I63.323, I63.329, I63.33, I63.331, I63.332, I63.333, I63.339, I63.34, I63.341, I63.342, I63.343, I63.349, I63.39, I63.40, I63.411, I63.412, I63.413, I63.419, I63.421, I63.422, I63.423, I63.429, I63.431, I63.432, I63.433, I63.439, I63.441, I63.442, I63.443, I63.449, I63.49, I63.5, I63.50, I63.51, I63.511, I63.512, I63.513, I63.519, I63.52, I63.521, I63.522, I63.523, I63.529, I63.53, I63.531, I63.532, I63.533, I63.539, I63.54, I63.541, I63.542, I63.543, I63.549, I63.59, I63.6, I63.8, I63.81, I63.89, I63.9
**MI**	410, 410.0, 410.00, 410.01, 410.02, 410.1, 410.10, 410.11, 410.12, 410.2, 410.20, 410.21, 410.22, 410.3, 410.30, 410.31, 410.32, 410.4, 410.40, 410.41, 410.42, 410.5, 410.50, 410.51, 410.52, 410.6, 410.60, 410.61, 410.62, 410.7, 410.70, 410.71, 410.72, 410.8, 410.80, 410.81, 410.82, 410.9, 410.90, 410.91, 410.92, I21, I21.0, I21.01, I21.02, I21.09, I21.1, I21.11, I21.19, I21.2, I21.21, I21.29, I21.3, I21.4, I21.9, I21.A1, I21.A9, I22, I22.0, I22.1, I22.2, I22.8, I22.9
**PCI/CABG**	36.2, 36.3, 36.30, 36.31, 36.32, 36.33, 36.34, 36.35, 36.36, 36.37, 36.38, 36.39, 3632, 3639
**Unstable Angina**	411.1, I20.0, I25.110, I25.700, I25.710, I25.720, I25.730, I25.750, I25.760, I25.790

Clinical condition	ICD code for clinical condition
**PCI**	0.66, 00.45, 00.46, 00.47, 00.48, 021K4Z8, 0270046, 027004Z, 0270056, 027005Z, 0270066, 027006Z, 0270076, 027007Z, 02700D6, 02700DZ, 02700EZ, 02700F6, 02700FZ, 02700G6, 02700GZ, 02700T6, 02700TZ, 02700Z6, 02700ZZ, 0270346, 027034Z, 0270356, 027035Z, 0270366, 027036Z, 0270376, 027037Z, 02703D6, 02703DZ, 02703E6, 02703EZ, 02703F6, 02703FZ, 02703G6, 02703GZ, 02703T6, 02703TZ, 02703Z6, 02703ZZ, 0270446, 027044Z, 0270456, 027045Z, 0270466, 027046Z, 0270476, 027047Z, 02704D6, 02704DZ, 02704E6, 02704EZ, 02704F6, 02704FZ, 02704G6, 02704GZ, 02704T6, 02704TZ, 02704Z6, 02704ZZ, 0271046, 027104Z, 0271056, 027105Z, 0271066, 027106Z, 0271076, 027107Z, 02710D6, 02710DZ, 02710EZ, 02710F6, 02710FZ, 02710G6, 02710GZ, 02710T6, 02710TZ, 02710Z6, 02710ZZ, 0271346, 027134Z, 0271356, 027135Z, 0271366, 027136Z, 0271376, 027137Z, 02713D6, 02713DZ, 02713E6, 02713EZ, 02713F6, 02713FZ, 02713G6, 02713GZ, 02713T6, 02713TZ, 02713Z6, 02713ZZ, 0271446, 027144Z, 0271456, 027145Z, 0271466, 027146Z, 0271476, 027147Z, 02714D6, 02714DZ, 02714E6, 02714EZ, 02714F6, 02714FZ, 02714G6, 02714GZ, 02714T6, 02714TZ, 02714Z6, 02714ZZ, 0272046, 027204Z, 0272056, 027205Z, 0272066, 027206Z, 0272076, 027207Z, 02720D6, 02720DZ, 02720EZ, 02720F6, 02720FZ, 02720G6, 02720GZ, 02720T6, 02720TZ, 02720Z6, 02720ZZ, 0272346, 027234Z, 0272356, 027235Z, 0272366, 027236Z, 0272376, 027237Z, 02723D6, 02723DZ, 02723E6, 02723EZ, 02723F6, 02723FZ, 02723G6, 02723GZ, 02723T6, 02723TZ, 02723Z6, 02723ZZ, 0272446, 027244Z, 0272456, 027245Z, 0272466, 027246Z, 0272476, 027247Z, 02724D6, 02724DZ, 02724E6, 02724EZ, 02724F6, 02724FZ, 02724G6, 02724GZ, 02724T6, 02724TZ, 02724Z6, 02724ZZ, 0273046, 027304Z, 0273056, 027305Z, 0273066, 027306Z, 0273076, 027307Z, 02730D6, 02730DZ, 02730EZ, 02730F6, 02730FZ, 02730G6, 02730GZ, 02730T6, 02730TZ, 02730Z6, 02730ZZ, 0273346, 027334Z, 0273356, 027335Z, 0273366, 027336Z, 0273376, 027337Z, 02733D6, 02733DZ, 02733E6, 02733EZ, 02733F6, 02733FZ, 02733G6, 02733GZ, 02733T6, 02733TZ, 02733Z6, 02733ZZ, 0273446, 027344Z, 0273456, 027345Z, 0273466, 027346Z, 0273476, 027347Z, 02734D6, 02734DZ, 02734E6, 02734EZ, 02734F6, 02734FZ, 02734G6, 02734GZ, 02734T6, 02734TZ, 02734Z6, 02734ZZ, 02C00Z6, 02C00ZZ, 02C03Z6, 02C03ZZ, 02C04Z6, 02C04ZZ, 02C10Z6, 02C10ZZ, 02C13Z6, 02C13ZZ, 02C14Z6, 02C14ZZ, 02C20Z6, 02C20ZZ, 02C23Z6, 02C23ZZ, 02C24Z6, 02C24ZZ, 02C30Z6, 02C30ZZ, 02C33Z6, 02C33ZZ, 02C34Z6, 02C34ZZ, 02H00DZ, 02H00YZ, 02H03DZ, 02H03YZ, 02H04DZ, 02H04YZ, 02H10DZ, 02H10YZ, 02H13DZ, 02H13YZ, 02H14DZ, 02H14YZ, 02H20DZ, 02H20YZ, 02H23DZ, 02H23YZ, 02H24DZ, 02H24YZ, 02H30DZ, 02H30YZ, 02H33DZ, 02H33YZ, 02H34DZ, 02H34YZ, 33140, 33141, 36.0, 36.00, 36.01, 36.02, 36.03, 36.04, 36.05, 36.06, 36.07, 36.08, 36.09, 3603, 3606, 3607, 3633, 3634, 92920, 92921, 92924, 92925, 92928, 92929, 92933, 92934, 92937, 92938, 92941, 92943, 92944, 92973, 92980, 92981, 92982, 92984, C1714, C1724, C1725, C1874, C1875, C1876, C1877, C1885, C2623, C9600, C9601, C9602, C9604, C9605, C9606, C9607, C9608, G0290, G0291

Clinical condition	ICD code for clinical condition
**CABG**	021008C, 021008F, 021008W, 0210093, 0210098, 0210099, 021009C, 021009F, 021009W, 02100A3, 02100A8, 02100A9, 02100AC, 02100AF, 02100AW, 02100J3, 02100J8, 02100J9, 02100JC, 02100JF, 02100JW, 02100K3, 02100K8, 02100K9, 02100KC, 02100KF, 02100KW, 02100Z3, 02100Z8, 02100Z9, 02100ZC, 02100ZF, 0210344, 02103D4, 0210444, 0210483, 0210488, 0210489, 021048C, 021048F, 021048W, 0210493, 0210498, 0210499, 021049C, 021049F, 021049W, 02104A3, 02104A8, 02104A9, 02104AC, 02104AF, 02104AW, 02104D4, 02104J3, 02104J8, 02104J9, 02104JC, 02104JF, 02104JW, 02104K3, 02104K8, 02104K9, 02104KC, 02104KF, 02104KW, 02104Z3, 02104Z8, 02104Z9, 02104ZC, 02104ZF, 0211083, 0211088, 0211089, 021108C, 021108F, 021108W, 0211093, 0211098, 0211099, 021109C, 021109F, 021109W, 02110A3, 02110A8, 02110A9, 02110AC, 02110AF, 02110AW, 02110J3, 02110J8, 02110J9, 02110JC, 02110JF, 02110JW, 02110K3, 02110K8, 02110K9, 02110KC, 02110KF, 02110KW, 02110Z3, 02110Z8, 02110Z9, 02110ZC, 02110ZF, 0211344, 02113D4, 0211444, 0211483, 0211488, 0211489, 021148C, 021148F, 021148W, 0211493, 0211498, 0211499, 021149C, 021149F, 021149W, 02114A3, 02114A8, 02114A9, 02114AC, 02114AF, 02114AW, 02114D4, 02114J3, 02114J8, 02114J9, 02114JC, 02114JF, 02114JW, 02114K3, 02114K8, 02114K9, 02114KC, 02114KF, 02114KW, 02114Z3, 02114Z8, 02114Z9, 02114ZC, 02114ZF, 0212083, 0212088, 0212089, 021208C, 021208F, 021208W, 0212093, 0212098, 0212099, 021209C, 021209F, 021209W, 02120A3, 02120A8, 02120A9, 02120AC, 02120AF, 02120AW, 02120J3, 02120J8, 02120J9, 02120JC, 02120JF, 02120JW, 02120K3, 02120K8, 02120K9, 02120KC, 02120KF, 02120KW, 02120Z3, 02120Z8, 02120Z9, 02120ZC, 02120ZF, 0212344, 02123D4, 0212444, 0212483, 0212488, 0212489, 021248C, 021248F, 021248W, 0212493, 0212498, 0212499, 021249C, 021249F, 021249W, 02124A3, 02124A8, 02124A9, 02124AC, 02124AF, 02124AW, 02124D4, 02124J3, 02124J8, 02124J9, 02124JC, 02124JF, 02124JW, 02124K3, 02124K8, 02124K9, 02124KC, 02124KF, 02124KW, 02124Z3, 02124Z8, 02124Z9, 02124ZC, 02124ZF, 0213083, 0213088, 0213089, 021308C, 021308F, 021308W, 0213093, 0213098, 0213099, 021309C, 021309F, 021309W, 02130A3, 02130A8, 02130A9, 02130AC, 02130AF, 02130AW, 02130J3, 02130J8, 02130J9, 02130JC, 02130JF, 02130JW, 02130K3, 02130K8, 02130K9, 02130KC, 02130KF, 02130KW, 02130Z3, 02130Z8, 02130Z9, 02130ZC, 02130ZF, 0213344, 02133D4, 0213444, 0213483, 0213488, 0213489, 021348C, 021348F, 021348W, 0213493, 0213498, 0213499, 021349C, 021349F, 021349W, 02134A3, 02134A8, 02134A9, 02134AC, 02134AF, 02134AW, 02134D4, 02134J3, 02134J8, 02134J9, 02134JC, 02134JF, 02134JW, 02134K3, 02134K8, 02134K9, 02134KC, 02134KF, 02134KW, 02134Z3, 02134Z8, 02134Z9, 02134ZC, 02134ZF, 33315, 33335, 33510, 33511, 33512, 33513, 33514, 33516, 33517, 33518, 33519, 33521, 33522, 33523, 33530, 33533, 33534, 33535, 33536, 33572, 33860, 33863, 33870, 36.1, 36.10, 36.11, 36.12, 36.13, 36.14, 36.15, 36.16, 36.17, 36.18, 36.19, 3610, 3611, 3612, 3613, 3614, 3615, 3616, 3619, 3631, S2205, S2206

Table S1c. Clinical conditions with associated ICD codes for comorbidities

Comorbidity	ICD Code for comorbidity
**Atrial fibrillation**	427.3, 427.31, I48.0, I48.1, I48.11, I48.19, I48.2, I48.20, I48.21, I48.91
**Chronic Kidney Disease**	285.21, 403.00, 403.01, 403.10, 403.11, 403.90, 403.91, 404.00, 404.01, 404.02, 404.03, 404.10, 404.11, 404.12, 404.13, 404.90, 404.91, 404.92, 404.93, 585.3, 585.4, 585.5, 585.6, 585.9, 646.2, D63.1, E08.22, E09.22, E10.22, E11.22, E13.22, I12.0, I13.11, I13.2, N18.3, N18.30, N18.31, N18.32, N18.4, N18.5, N18.6, N18.9, O10.211, O10.212, O10.213, O10.219, O10.22, O10.23, O10.311, O10.312, O10.313, O10.319, O10.32, O10.33
**Diabetes**	249.00, 249.01, 249.10, 249.11, 249.20, 249.21, 249.30, 249.31, 249.40, 249.41, 249.50, 249.51, 249.60, 249.61, 249.70, 249.71, 249.80, 249.81, 249.90, 249.91, 250, 250.0, 250.00, 250.01, 250.02, 250.03, 250.1, 250.10, 250.11, 250.12, 250.13, 250.2, 250.20, 250.21, 250.22, 250.23, 250.3, 250.30, 250.31, 250.32, 250.33, 250.4, 250.40, 250.41, 250.42, 250.43, 250.5, 250.50, 250.51, 250.52, 250.53, 250.6, 250.60, 250.61, 250.62, 250.63, 250.7, 250.70, 250.71, 250.72, 250.73, 250.8, 250.80, 250.81, 250.82, 250.83, 250.9, 250.90, 250.91, 250.92, 250.93, 357.2, E08.00, E08.01, E08.10, E08.11, E08.21, E08.22, E08.29, E08.311, E08.319, E08.321, E08.3211, E08.3212, E08.3213, E08.3219, E08.329, E08.3291, E08.3292, E08.3293, E08.3299, E08.331, E08.3311, E08.3312, E08.3313, E08.3319, E08.339, E08.3391, E08.3392, E08.3393, E08.3399, E08.341, E08.3411, E08.3412, E08.3413, E08.3419, E08.349, E08.3491, E08.3492, E08.3493, E08.3499, E08.351, E08.3511, E08.3512, E08.3513, E08.3519, E08.3521, E08.3522, E08.3523, E08.3529, E08.3531, E08.3532, E08.3533, E08.3539, E08.3541, E08.3542, E08.3543, E08.3549, E08.3551, E08.3552, E08.3553, E08.3559, E08.359, E08.3591, E08.3592, E08.3593, E08.3599, E08.36, E08.37X1, E08.37X2, E08.37X3, E08.37X9, E08.39, E08.40, E08.41, E08.42, E08.43, E08.44, E08.49, E08.51, E08.52, E08.59, E08.610, E08.618, E08.620, E08.621, E08.622, E08.628, E08.630, E08.638, E08.641, E08.649, E08.65, E08.69, E08.8, E08.9

Comorbidity	ICD Code for comorbidity
**Diabetes (continued)**	E09.00, E09.01, E09.10, E09.11, E09.21, E09.22, E09.29, E09.311, E09.319, E09.321, E09.3211, E09.3212, E09.3213, E09.3219, E09.329, E09.3291, E09.3292, E09.3293, E09.3299, E09.331, E09.3311, E09.3312, E09.3313, E09.3319, E09.339, E09.3391, E09.3392, E09.3393, E09.3399, E09.341, E09.3411, E09.3412, E09.3413, E09.3419, E09.349, E09.3491, E09.3492, E09.3493, E09.3499, E09.351, E09.3511, E09.3512, E09.3513, E09.3519, E09.3521, E09.3522, E09.3523, E09.3529, E09.3531, E09.3532, E09.3533, E09.3539, E09.3541, E09.3542, E09.3543, E09.3549, E09.3551, E09.3552, E09.3553, E09.3559, E09.359, E09.3591, E09.3592, E09.3593, E09.3599, E09.36, E09.37X1, E09.37X2, E09.37X3, E09.37X9, E09.39, E09.40, E09.41, E09.42, E09.43, E09.44, E09.49, E09.51, E09.52, E09.59, E09.610, E09.618, E09.620, E09.621, E09.622, E09.628, E09.630, E09.638, E09.641, E09.649, E09.65, E09.69, E09.8, E09.9, E10.10, E10.11, E10.21, E10.22, E10.29, E10.311, E10.319, E10.321, E10.3211, E10.3212, E10.3213, E10.3219, E10.329, E10.3291, E10.3292, E10.3293, E10.3299, E10.331, E10.3311, E10.3312, E10.3313, E10.3319, E10.339, E10.3391, E10.3392, E10.3393, E10.3399, E10.341, E10.3411, E10.3412, E10.3413, E10.3419, E10.349, E10.3491, E10.3492, E10.3493, E10.3499, E10.351, E10.3511, E10.3512, E10.3513, E10.3519, E10.3521, E10.3522, E10.3523, E10.3529, E10.3531, E10.3532, E10.3533, E10.3539, E10.3541, E10.3542, E10.3543, E10.3549, E10.3551, E10.3552, E10.3553, E10.3559, E10.359, E10.3591, E10.3592, E10.3593, E10.3599, E10.36, E10.37X1, E10.37X2, E10.37X3, E10.37X9, E10.39, E10.40, E10.41, E10.42, E10.43, E10.44, E10.49, E10.51, E10.52, E10.59, E10.610, E10.618, E10.620, E10.621, E10.622, E10.628, E10.630, E10.638, E10.641, E10.649, E10.65, E10.69, E10.8, E10.9, E11.00, E11.01, E11.10, E11.11, E11.21, E11.22, E11.29, E11.311, E11.319, E11.321, E11.3211, E11.3212, E11.3213, E11.3219, E11.329, E11.3291, E11.3292, E11.3293, E11.3299, E11.331, E11.3311, E11.3312, E11.3313, E11.3319, E11.339, E11.3391, E11.3392, E11.3393, E11.3399, E11.341, E11.3411, E11.3412, E11.3413, E11.3419, E11.349, E11.3491, E11.3492, E11.3493, E11.3499, E11.351, E11.3511, E11.3512, E11.3513, E11.3519, E11.3521, E11.3522, E11.3523, E11.3529, E11.3531, E11.3532, E11.3533, E11.3539, E11.3541, E11.3542, E11.3543, E11.3549, E11.3551, E11.3552, E11.3553, E11.3559, E11.359, E11.3591, E11.3592, E11.3593, E11.3599, E11.36, E11.37X1, E11.37X2, E11.37X3, E11.37X9, E11.39, E11.40, E11.41, E11.42, E11.43, E11.44, E11.49, E11.51, E11.52, E11.59, E11.610, E11.618, E11.620, E11.621, E11.622, E11.628, E11.630, E11.638, E11.641, E11.649, E11.65, E11.69, E11.8, E11.9

Comorbidity	ICD Code for comorbidity
**Diabetes (concluded)**	E13.00, E13.01, E13.10, E13.11, E13.21, E13.22, E13.29, E13.311, E13.319, E13.321, E13.3211, E13.3212, E13.3213, E13.3219, E13.329, E13.3291, E13.3292, E13.3293, E13.3299, E13.331, E13.3311, E13.3312, E13.3313, E13.3319, E13.339, E13.3391, E13.3392, E13.3393, E13.3399, E13.341, E13.3411, E13.3412, E13.3413, E13.3419, E13.349, E13.3491, E13.3492, E13.3493, E13.3499, E13.351, E13.3511, E13.3512, E13.3513, E13.3519, E13.3521, E13.3522, E13.3523, E13.3529, E13.3531, E13.3532, E13.3533, E13.3539, E13.3541, E13.3542, E13.3543, E13.3549, E13.3551, E13.3552, E13.3553, E13.3559, E13.359, E13.3591, E13.3592, E13.3593, E13.3599, E13.36, E13.37X1, E13.37X2, E13.37X3, E13.37X9, E13.39, E13.40, E13.41, E13.42, E13.43, E13.44, E13.49, E13.51, E13.52, E13.59, E13.610, E13.618, E13.620, E13.621, E13.622, E13.628, E13.630, E13.638, E13.641, E13.649, E13.65, E13.69, E13.8, E13.9, O24.011, O24.012, O24.013, O24.019, O24.02, O24.03, O24.111, O24.112, O24.113, O24.119, O24.12, O24.13, O24.311, O24.312, O24.313, O24.319, O24.32, O24.33, O24.811, O24.812, O24.813, O24.819, O24.82, O24.83
**Hyperlipidemia**	272.0, 272.1, 272.2, 272.3, 272.4, 272.7, 272.8, 272.9, E78.0, E78.00, E78.01, E78.1, E78.2, E78.3, E78.4, E78.49, E78.5, E78.89, E78.9
**Hypertension**	360.42, 362.11, 401, 401.0, 401.1, 401.9, 402, 402.0, 402.00, 402.01, 402.1, 402.10, 402.11, 402.9, 402.90, 402.91, 403, 403.0, 403.00, 403.01, 403.1, 403.10, 403.11, 403.9, 403.90, 403.91, 404, 404.0, 404.00, 404.01, 404.02, 404.03, 404.1, 404.10, 404.11, 404.12, 404.13, 404.9, 404.90, 404.91, 404.92, 404.93, 405, 405.0, 405.01, 405.09, 405.1, 405.11, 405.19, 405.9, 405.91, 405.99, 437.2, 642.2, 642.20, 642.21, 642.22, 642.23, 642.24, 642.7, 642.70, 642.71, 642.72, 642.73, 642.74, 997.91, H35.031, H35.032, H35.033, H35.039, I10, I11.0, I11.9, I12.0, I12.9, I13.0, I13.10, I13.11, I13.2, I15.0, I15.1, I15.2, I15.8, I15.9, I16.0, I16.1, I16.9, I67.4, O10.011, O10.012, O10.013, O10.019, O10.02, O10.03, O10.111, O10.112, O10.113, O10.119, O10.12, O10.13, O10.211, O10.212, O10.213, O10.219, O10.22, O10.23, O10.311, O10.312, O10.313, O10.319, O10.32, O10.33, O10.411, O10.412, O10.413, O10.419, O10.42, O10.43, O10.911, O10.912, O10.913, O10.919, O10.92, O10.93, O11.1, O11.2, O11.3, O11.4, O11.5, O11.9
