# Supplementary material for: Lipoprotein(a) and recurrent atherosclerotic cardiovascular events: the US Family Heart Database
Source: Eur Heart J. 2025 May 7;46(44):4762–75. doi: 10.1093/eurheartj/ehaf297 (PMC12634116; doi:10.1093/eurheartj/ehaf297)
Supplement: ehaf297_Supplementary_Data [file ehaf297_supplementary_data.zip › supp_table2.pdf]

Table S2. Covariate characteristics by lipoprotein(a) category

	Lipoprotein(a) Category (nmol/L)					
	< 15<br>< 33%<br>(N= 85,025)	15 to 79<br>33% to 66%<br>(N= 97,958)	80 to 179<br>67% to 84%<br>(N= 47,240)	180 to 299<br>85% to 94%<br>(N= 29,541)	>= 300<br>>= 95%<br>(N= 14,006)	Total<br>(N=273,770)
**Age (yrs), n (%)**						
< 50	11,124 (32)	12,147 (35)	6,486 (18)	3,737 (11)	1,632 (5)	35,126 (100)
50-59	19,819 (31)	21,721 (34)	11,272 (18)	7,105 (11)	3,427 (5)	63,344 (100)
60-69	30,611 (31)	34,763 (35)	16,795 (17)	10,851 (11)	5,324 (5)	98,344 (100)
>= 70	23,471 (30)	29,327 (38)	12,687 (16)	7,848 (10)	3,623 (5)	76,956 (100)
**Women, n (%)**	32,400 (28)	42,625 (36)	20,524 (18)	14,208 (12)	7,512 (6)	117,269 (100)
**Race/Ethnicity, n (%)**						
Black Individuals	3,895 (17)	7,281 (32)	5,691 (25)	3,510 (16)	2,074 (9)	22,451 (100)
Hispanic Individuals	8,276 (34)	9,216 (37)	3,919 (16)	2,233 (9)	962 (4)	24,606 (100)
White Individuals	53,515 (33)	56,831 (35)	26,270 (16)	16,898 (10)	7,651 (5)	161,165 (100)
Other Individuals	3,651 (29)	5,298 (41)	2,154 (17)	1,145 (9)	537 (4)	12,785 (100)
Unknown	15,688 (30)	19,332 (37)	9,206 (17)	5,755 (11)	2,782 (5)	52,763 (100)
**Education Level, n (%)**						
High school grad or less	14,224 (30)	16,535 (35)	8,708 (18)	5,529 (12)	2,773 (6)	47,769 (100)
Some college	27,793 (32)	30,440 (35)	14,873 (17)	9,466 (11)	4,434 (5)	87,006 (100)
Assoc/Bachelor degree plus	26,480 (32)	30,816 (37)	13,996 (17)	8,503 (10)	3,882 (5)	83,677 (100)
Unknown	16,528 (30)	20,167 (36)	9,663 (17)	6,043 (11)	2,917 (5)	55,318 (100)
**Household Income, n (%)**						
<$30K	9,447 (28)	11,879 (35)	6,503 (19)	4,155 (12)	2,123 (6)	34,107 (100)
$30-49K	8,320 (30)	9,846 (35)	4,958 (18)	3,086 (11)	1,562 (6)	27,772 (100)
$50-74K	12,338 (31)	13,857 (35)	6,821 (17)	4,275 (11)	2,053 (5)	39,344 (100)
$75-99K	11,473 (32)	12,639 (36)	5,870 (17)	3,724 (11)	1,662 (5)	35,368 (100)
$100K+	22,787 (33)	24,767 (36)	11,460 (17)	7,066 (10)	3,213 (5)	69,293 (100)
Unknown	20,660 (30)	24,970 (37)	11,628 (17)	7,235 (11)	3,393 (5)	67,886 (100)
**US region, n (%)**						
Midwest	8,552 (32)	9,106 (34)	4,622 (17)	2,968 (11)	1,361 (5)	26,609 (100)
Northeast	15,885 (29)	20,601 (37)	9,629 (17)	6,127 (11)	3,038 (5)	55,280 (100)
South	39,916 (31)	44,592 (35)	22,345 (18)	13,711 (11)	6,534 (5)	127,098 (100)
West	17,408 (32)	19,860 (37)	8,802 (16)	5,497 (10)	2,521 (5)	54,088 (100)
Unknown	3,264 (31)	3,799 (36)	1,842 (17)	1,238 (12)	552 (5)	10,695 (100)
**Hyperlipidemia, n (%)**	71,415 (31)	82,822 (36)	40,390 (17)	25,947 (11)	12,593 (5)	233,167 (100)
**Chronic Kidney Disease, n (%)**	7,900 (27)	10,558 (37)	5,298 (18)	3,300 (11)	1,712 (6)	28,768 (100)
**Diabetes, n (%)**	28,318 (31)	31,680 (35)	15,586 (17)	9,508 (11)	4,979 (6)	90,071 (100)
**Atrial Fibrillation, n (%)**	10,701 (32)	12,203 (37)	5,491 (17)	3,320 (10)	1,398 (4)	33,113 (100)
**Charlson Comorbidity Index, n (%)**						
0	38,501 (31)	43,557 (35)	21,363 (17)	13,601 (11)	6,263 (5)	123,285 (100)
1-2	28,866 (31)	33,366 (36)	16,000 (17)	9,906 (11)	4,787 (5)	92,925 (100)
3+	17,658 (31)	21,035 (37)	9,877 (17)	6,034 (10)	2,956 (5)	57,560 (100)
**Lipid-lowering therapy, n (%)**						
PCSK9i Mono/Combo	1,051 (28)	1,076 (28)	732 (19)	566 (15)	356 (9)	3,781 (100)
Eze/BA w/Statins	4,114 (26)	5,040 (31)	2,881 (18)	2,412 (15)	1,596 (10)	16,043 (100)
Statins-High Intensity	19,711 (30)	22,846 (34)	11,703 (18)	8,182 (12)	4,226 (6)	66,668 (100)
Statins-Low/Med Intensity	22,749 (32)	25,887 (37)	12,047 (17)	7,129 (10)	2,985 (4)	70,797 (100)
Eze and/or BA	811 (31)	908 (34)	477 (18)	313 (12)	129 (5)	2,638 (100)
None	36,589 (32)	42,201 (37)	19,400 (17)	10,939 (10)	4,714 (4)	113,843 (100)
**Duration of LLT - n (%)**						
LLT Use > 75%	28,097 (30)	32,114 (35)	15,720 (17)	10,885 (12)	5,532 (6)	92,348 (100)
<=75%/None	56,928 (31)	65,844 (36)	31,520 (17)	18,656 (10)	8,474 (5)	181,422 (100)
**Hypertension/Meds - n (%)**						
Hypertension/Meds	43,954 (31)	50,577 (35)	24,678 (17)	15,863 (11)	7,874 (6)	142,946 (100)
Hypertension/No Meds	19,680 (31)	22,941 (36)	10,933 (17)	6,615 (10)	3,091 (5)	63,260 (100)
No Hypertension	21,391 (32)	24,440 (36)	11,629 (17)	7,063 (10)	3,041 (5)	67,564 (100)
**Anti-Platelet Meds, n (%)**	18,857 (29)	22,091 (34)	11,666 (18)	7,822 (12)	4,139 (6)	64,575 (100)

	Lipoprotein(a) Category (nmol/L)					
	< 15<br>< 33%<br>(N= 85,025)	15 to 79<br>33% to 66%<br>(N= 97,958)	80 to 179<br>67% to 84%<br>(N= 47,240)	180 to 299<br>85% to 94%<br>(N= 29,541)	>= 300<br>>= 95%<br>(N= 14,006)	Total<br>(N=273,770)
**Baseline ASCVD - n (%)**						
Coronary	57,274 (31)	65,024 (35)	31,834 (17)	20,432 (11)	9,901 (5)	184,465 (100)
Cerebral	16,762 (31)	19,890 (37)	9,380 (17)	5,601 (10)	2,474 (5)	54,107 (100)
Peripheral	9,600 (31)	11,624 (37)	5,372 (17)	3,106 (10)	1,487 (5)	31,189 (100)
Unknown	1,389 (35)	1,420 (35)	654 (16)	402 (10)	144 (4)	4,009 (100)
**Laboratory Values, n (%)**						
**Total Cholesterol (mg/dL)**						
< 120	9,203 (36)	9,436 (37)	4,146 (16)	2,085 (8)	720 (3)	25,590 (100)
120-159	23,820 (31)	27,373 (36)	12,881 (17)	8,144 (11)	3,747 (5)	75,965 (100)
160-199	17,679 (30)	21,159 (36)	10,247 (17)	6,645 (11)	3,571 (6)	59,301 (100)
200-239	8,249 (28)	10,580 (37)	5,269 (18)	3,230 (11)	1,655 (6)	28,983 (100)
>= 240	3,340 (26)	4,603 (36)	2,248 (18)	1,557 (12)	913 (7)	12,661 (100)
Missing	22,734 (32)	24,807 (35)	12,449 (17)	7,880 (11)	3,400 (5)	71,270 (100)
**LDL-C (mg/dL)**						
< 60	13,527 (36)	13,794 (37)	5,898 (16)	3,095 (8)	1,111 (3)	37,425 (100)
60-79	13,872 (30)	16,794 (36)	7,874 (17)	5,182 (11)	2,428 (5)	46,150 (100)
80-99	10,328 (29)	12,398 (35)	5,959 (17)	4,056 (12)	2,296 (7)	35,037 (100)
100-119	6,902 (29)	8,610 (37)	4,132 (18)	2,492 (11)	1,439 (6)	23,575 (100)
>= 120	8,625 (27)	11,910 (38)	5,809 (18)	3,524 (11)	1,844 (6)	31,712 (100)
Missing	31,771 (32)	34,452 (34)	17,568 (18)	11,192 (11)	4,888 (5)	99,871 (100)
**Triglycerides (mg/dL)**						
< 100	23,932 (28)	31,099 (36)	16,203 (19)	9,626 (11)	4,537 (5)	85,397 (100)
100-149	19,182 (30)	23,379 (37)	10,687 (17)	7,092 (11)	3,540 (6)	63,880 (100)
150-199	9,564 (33)	10,433 (36)	4,491 (16)	2,872 (10)	1,479 (5)	28,839 (100)
200-249	4,381 (37)	4,194 (35)	1,710 (14)	1,097 (9)	557 (5)	11,939 (100)
>= 250	4,977 (43)	3,737 (32)	1,535 (13)	882 (8)	443 (4)	11,574 (100)
Missing	22,989 (32)	25,116 (35)	12,614 (17)	7,972 (11)	3,450 (5)	72,141 (100)

Comorbidities were assessed from the earliest activity in the database to 365 days after the first diagnosis of atherosclerotic cardiovascular disease. Medication use was determined from 8 to 365 days after the first diagnosis of atherosclerotic cardiovascular disease. Individuals were included in only one baseline ASCVD category tiered by coronary followed by cerebrovascular. LDL cholesterol, total cholesterol, and triglyceride values were median of values within 60 days after the first diagnosis of atherosclerotic cardiovascular disease through end of follow-up. PCSK9i = proprotein convertase subtilisin/kexin type 9 inhibitors, BA=bempedoic acid; eze= ezetimibe; LDL = low density lipoprotein; yr=year.
