# Supplementary material for: Lipoprotein(a) and recurrent atherosclerotic cardiovascular events: the US Family Heart Database
Source: Eur Heart J. 2025 May 7;46(44):4762–75. doi: 10.1093/eurheartj/ehaf297 (PMC12634116; doi:10.1093/eurheartj/ehaf297)
Supplement: ehaf297_Supplementary_Data [file ehaf297_supplementary_data.zip › supp_table3.pdf]

Table S3. Demographic characteristics for each lipoprotein category: Women

Lipoprotein(a) Category (nmol/L)					
	<15<br><33%<br>N=85,025	15 to 79<br>33% to 66%<br>N=97,958	80 to 179<br>67% to 84%<br>N=47,240	180 to 299<br>85% to 94%<br>N=29,541	≥300<br>≥95%<br>N=14,006
**Female, n (%)**	32,400 (38.1)	42,625 (43.5)	20,524 (43.4)	14,208 (48.1)	7,512 (53.6)
**Age (yr)**	65 (56–71)	65 (56–71)	64 (55–70)	65 (56–70)	64 (57–70)
**Race/Ethnicity, n (%)**					
Black	1,685 (5)	3,700 (9)	3,161 (15)	2,075 (15)	1,332 (18)
Hispanic	3,411 (11)	4,329 (10)	1,789 (9)	1,130 (8)	539 (7)
White	19,288 (60)	23,406 (55)	10,384 (51)	7,605 (54)	3,794 (51)
Other	1,189 (4)	1,747 (4)	707 (3)	427 (3)	249 (3)
Unknown	6,827 (21)	9,443 (22)	4,483 (22)	2,971 (21)	1,598 (21)
**Charlson Comorbidity Index, n (%)**					
0	13,042 (40)	17,098 (40)	8,262 (40)	5,833 (41)	3,007 (40)
1–2	11,643 (36)	15,430 (36)	7,346 (36)	5,112 (36)	2,730 (36)
3+	7,715 (24)	10,097 (24)	4,916 (24)	3,263 (23)	1,775 (24)
**Risk factors, n (%)**					
Hypertension	23,468 (72)	31,503 (74)	15,413 (75)	10,855 (76)	5,919 (79)
Diabetes	10,622 (33)	13,786 (32)	7,062 (34)	4,771 (34)	2,771 (37)
Familial Hypercholesterolemia	261 (0.8)	398 (0.9)	202 (1.0)	186 (1.3)	104 (1.4)
**Lipid-lowering therapy n (%)**	16,381 (51)	21,974 (52)	10,985 (54)	8,372 (59)	4,813 (64)
**Laboratory values**					
Lipoprotein(a) (nmol/L)	9.9 (9.9–10.0)	34.0 (23.0–51.0)	128.0 (102.0–155.0)	218.0 (195.0–254.0)	370.0 (331.0–437.0)
LDL cholesterol (mg/dL)	89.0 (67.5–117.0)	92.0 (70.0–121.0)	94.0 (72.5–122.0)	93.0 (72.5–121.5)	95.5 (77.0–121.0)
Triglycerides (mg/dL)	115.0 (84.0–161.0)	110.0 (81.5–150.5)	104.0 (77.5–143.0)	107.0 (80.0–143.0)	107.0 (81.0–147.0)

Lipoprotein(a) and laboratory values are presented as median (interquartile range). Categorical variables are displayed as frequency (%). LDL = low density lipoprotein; yr = year.
