# Supplementary material for: Lipoprotein(a) and recurrent atherosclerotic cardiovascular events: the US Family Heart Database
Source: Eur Heart J. 2025 May 7;46(44):4762–75. doi: 10.1093/eurheartj/ehaf297 (PMC12634116; doi:10.1093/eurheartj/ehaf297)
Supplement: ehaf297_Supplementary_Data [file ehaf297_supplementary_data.zip › supp_table5.pdf]

Table S5. Demographic characteristics for each lipoprotein category: Black individuals

Lipoprotein(a) Category (nmol/L)					
	<15<br><33%<br>N=85,025	15 to 79<br>33% to 66%<br>N=97,958	80 to 179<br>67% to 84%<br>N=47,240	180 to 299<br>85% to 94%<br>N=29,541	≥300<br>≥95%<br>N=14,006
**Race/Ethnicity Black, n (%)**	3,895 (4.6)	7,281 (7.4)	5,691 (12.0)	3,510 (11.9)	2,074 (14.8)
**Age (yr)**	62 (54–69)	62 (53–69)	61 (52–69)	62 (53–69)	62 (54–69)
**Female, n (%)**	1,685 (43)	3,700 (51)	3,161 (56)	2,075 (59)	1,332 (64)
**Charlson Comorbidity Index, n (%)**					
0	1,490 (38)	2,498 (34)	1,969 (35)	1,174 (33)	698 (34)
1–2	1,412 (36)	2,667 (37)	2,041 (36)	1,288 (37)	748 (36)
3+	993 (25)	2,116 (29)	1,681 (30)	1,048 (30)	628 (30)
**Risk factors, n (%)**					
Hypertension	3,186 (82)	6,174 (85)	4,945 (87)	3,042 (87)	1,852 (89)
Diabetes	1,612 (41)	3,214 (44)	2,659 (47)	1,670 (48)	1,091 (53)
Familial Hypercholesterolemia	26 (0.7)	35 (0.5)	25 (0.4)	29 (0.8)	12 (0.6)
**Lipid-lowering therapy n (%)**	2,209 (57)	4,017 (55)	3,331 (59)	2,142 (61)	1,380 (67)
**Laboratory values**					
Lipoprotein(a) (nmol/L)	9.9<br>(7.0–10.0)	42.0<br>(26.0–59.0)	127.0<br>(101.0–154.0)	218.0<br>(195.0–254.0)	376.0<br>(332.0–465.0)
LDL cholesterol (mg/dL)	80.5<br>(60.0–106.0)	85.0<br>(65.0–112.0)	90.0<br>(69.0–116.0)	90.0<br>(71.0–118.0)	98.0<br>(78.0–123.0)
Triglycerides (mg/dL)	118.0<br>(85.5–170.0)	102.0<br>(75.5–143.0)	97.0<br>(72.5–131.0)	99.5<br>(75.0–136.5)	100.0<br>(76.0–136.0)

Lipoprotein(a) and laboratory values are presented as median (interquartile range). Categorical variables are displayed as frequency (%). LDL = low density lipoprotein; yr, year.
