# Supplementary material for: Lipoprotein(a) and recurrent atherosclerotic cardiovascular events: the US Family Heart Database
Source: Eur Heart J. 2025 May 7;46(44):4762–75. doi: 10.1093/eurheartj/ehaf297 (PMC12634116; doi:10.1093/eurheartj/ehaf297)
Supplement: ehaf297_Supplementary_Data [file ehaf297_supplementary_data.zip › supp_table8.pdf]

Table S8. Sensitivity analysis: risk of recurrent ASCVD events (unadjusted) by lipoprotein(a) categories: <75 nmol/L, 75–124 nmol/L, 125–199 nmol/L, 200–399 nmol/L and ≥400 nmol/L

Lipoprotein(a) category (nmol/L)	n	n with events	HR (95% CI)	p-value
<75	179,719	25,473	1	–
75–124	25,509	4,049	1.12 (1.08, 1.16)	3×10^{-11}
125–199	34,955	5,823	1.17 (1.14, 1.20)	10×10^{-27}
200–399	28,943	5,350	1.30 (1.26, 1.33)	3×10^{-66}
≥400	4,644	992	1.49 (1.40, 1.59)	9×10^{-35}

ASCVD = atherosclerotic cardiovascular disease; CI = confidence interval; HR = hazard ratio.
